# Human osteoblast and fibroblast response to oral implant biomaterials functionalized with non-thermal oxygen plasma

**DOI:** 10.1038/s41598-021-96526-x

**Published:** 2021-08-27

**Authors:** Kerstin Rabel, Ralf-Joachim Kohal, Thorsten Steinberg, Bernd Rolauffs, Erik Adolfsson, Brigitte Altmann

**Affiliations:** 1 Department of Prosthetic Dentistry, Center for Dental Medicine, Medical Center - University of Freiburg, Faculty of Medicine, University of Freiburg, Hugstetterstr. 55, 79106 Freiburg, Germany; 2 Department of Oral Biotechnology, Center for Dental Medicine, Medical Center - University of Freiburg, Faculty of Medicine, University of Freiburg, Hugstetterstr. 55, 79106 Freiburg, Germany; 3 G.E.R.N. Center for Tissue Replacement, Regeneration & Neogenesis, Department of Orthopedics and Trauma Surgery, Medical Center - University of Freiburg, Faculty of Medicine, University of Freiburg, Engesserstr. 4, 79108 Freiburg, Germany; 4 RISE IVF AB, RISE Research Institutes of Sweden, Argongatan 30, 43153 Mölndal, Sweden; 5 G.E.R.N. Center for Tissue Replacement, Regeneration & Neogenesis, Department of Prosthetic Dentistry, Medical Center - University of Freiburg, Faculty of Medicine, University of Freiburg, Engesserstr. 4, 79108 Freiburg, Germany

**Keywords:** Preclinical research, Implants, Biomaterials - cells

## Abstract

Plasma-treatment of oral implant biomaterials prior to clinical insertion is envisaged as a potential surface modification method for enhanced implant healing. To investigate a putative effect of plasma-functionalized implant biomaterials on oral tissue cells, this investigation examined the response of alveolar bone osteoblasts and gingival fibroblasts to clinically established zirconia- and titanium-based implant surfaces for bone and soft tissue integration. The biomaterials were either functionalized with oxygen-plasma in a plasma-cleaner or left untreated as controls, and were characterized in terms of topography and wettability. For the biological evaluation, the cell adhesion, morphogenesis, metabolic activity and proliferation were examined, since these parameters are closely interconnected during cell-biomaterial interaction. The results revealed that plasma-functionalization increased implant surface wettability. The magnitude of this effect thereby depended on surface topography parameters and initial wettability of the biomaterials. Concerning the cell response, plasma-functionalization of smooth surfaces affected initial fibroblast morphogenesis, whereas osteoblast morphology on rough surfaces was mainly influenced by topography. The plasma- and topography-induced differential cell morphologies were however not strong enough to trigger a change in proliferation behaviour. Hence, the results indicate that oxygen plasma-functionalization represents a possible cytocompatible implant surface modification method which can be applied for tailoring implant surface wettability.

## Introduction

In contrast to orthopaedic bone implants, oral implants not only need to integrate into the surrounding bone but also into the adjacent soft tissues to achieve a long-term clinical implant survival^1,2^. This is due to the fact that two components are necessary for an implant-supported replacement of teeth: (1) an endosseous implant part placed into the jawbone and (2) a transgingival part piercing the oral mucosa and allowing for the anchorage of the prosthetic restoration, such as a crown^2,3^. In this context, it is widely accepted that the cellular functions at the implant-tissue interface and thus the tissue integration process are influenced by the topographical and physicochemical properties of the biomaterial surface^2,3^. The optimization of the tissue integration process via modification of topographical and physicochemical surface characteristics advanced since the 1980s and led up to now to tissue-adopted biomaterials with improved surface finish. Regarding this issue, rough endosseous implant surfaces better support bone tissue integration^4^, whereas smooth surfaces are favourable for the soft tissue integration of the transgingival implant part, also termed abutment^5–7^.

However, despite improved surface finish, bacterial invasion from the oral cavity to the implant-tissue interface can compromise successful healing and long-term stability of oral implants^2,3,8^. In order to create optimized conditions for implant healing, surface modification methods that aim at reducing biofilm formation whilst at the same time promoting the cell-mediated tissue integration have come to the fore^9,10^. One possible surface modification method considered in this context is the functionalization of oral implant biomaterials with non-thermal gas plasmas^9,10^. This procedure involves the exposition of oral implants and/ or their abutments to non-thermal gas plasmas prior to clinical insertion^11^. Since gas plasmas can be generated by commercially available plasma-cleaners or plasma-jets, this process could be integrated into clinical practice as chairside surface modification method^11–14^. In order to prevent biofilm formation on biomaterials, non-thermal gas plasmas based on oxygen, argon, air or ammonia can be applied^9,13,15–19^. The response of implant relevant target-cells, namely bone forming cells and fibroblasts of the gingival soft tissues, was characterized on titanium and zirconia biomaterials exposed to non-thermal gas-plasmas composed of argon, oxygen as well as air. In detail, the response of bone-forming osteoblast-like cells was evaluated on biomaterials functionalized with argon and/or oxygen plasma^11,20–28^, whereas the fibroblast response further was characterized on surfaces functionalized with air-plasma^29–32^. The direct comparison of biomaterials functionalized with either argon or oxygen performed by Smeets et al.^27^ and Guo et al.^29^ revealed that oxygen plasma-functionalization better supported the attachment and proliferation of bone-forming osteoblast-like cells and gingival fibroblasts compared to the functionalization with argon plasma. Hitherto, investigations analysing the effects of oral implant functionalization with oxygen plasma on bone-forming osteoblast-like cells employed the human osteosarcoma cell line MG-63 and the murine osteoblastic cell line MC3T3-E1^11,23,27^. However, it has been demonstrated that MG-63 and MC3T3-E1 cells can differ in their response to surface characteristics from primary human osteoblasts^33–36^, which are the protagonists of oral implant integration into human bone. In the context of oral implant functionalization, the authors observed in a previous investigation^37^ that primary human osteoblasts were less sensitive to UV-functionalization of implant materials than described by other studies using MG-63 and MC3T3-E1 cells^11,38,39^. With respect to the interaction of fibroblasts with oxygen plasma-functionalized implant surfaces, there is—to the best of our knowledge—only one investigation characterizing the response of gingival fibroblasts in the very early period of 48 h after cell seeding on the biomaterials^29^. Hence, it is unknown whether oxygen plasma-functionalization affects the gingival fibroblast response beyond this very early time point.

Therefore, the objective of this study was to examine the cell response of primary human alveolar bone osteoblasts (AO) and gingival fibroblasts (GF) to oxygen plasma-functionalized implant surfaces over a period of 7 days. The null hypothesis of this study was that the response of primary human osteoblasts and gingival fibroblasts concerning attachment, morphogenesis and proliferation to plasma-functionalized biomaterial surfaces is comparable to untreated controls. Three currently established oral implant biomaterials, namely alumina-toughened zirconia (ATZ), yttria-stabilized tetragonal zirconia polycrystals (Y-TZP) and titanium (Ti) with commercially available optimized surfaces for enhanced bone and soft tissue integration served as cell culture substrates. The functionalization with oxygen plasma was performed in a commercially available plasma-cleaner. Biomaterial surfaces were characterized with regard to surface topography and wettability. For the biological evaluation the authors examined the cell response of AO and GF to plasma-treated and untreated control surfaces in terms of cell adhesion, cell morphogenesis, cell metabolic activity and proliferation. These cell parameters are known to play an important role during initial cell-biomaterial interaction^37,40–42^. In this context, the authors recently demonstrated that AO proliferation on ceramic oral implant surfaces is a function of cell shape/area during the first week of cell culture and by this revealed AO morphology as sensitive biomarker for transmitting implant surface cues into the cellular response^43^.

## Results

### Surface characterization

#### Topography of roughened surfaces for osteoblasts

**Figure 1:**
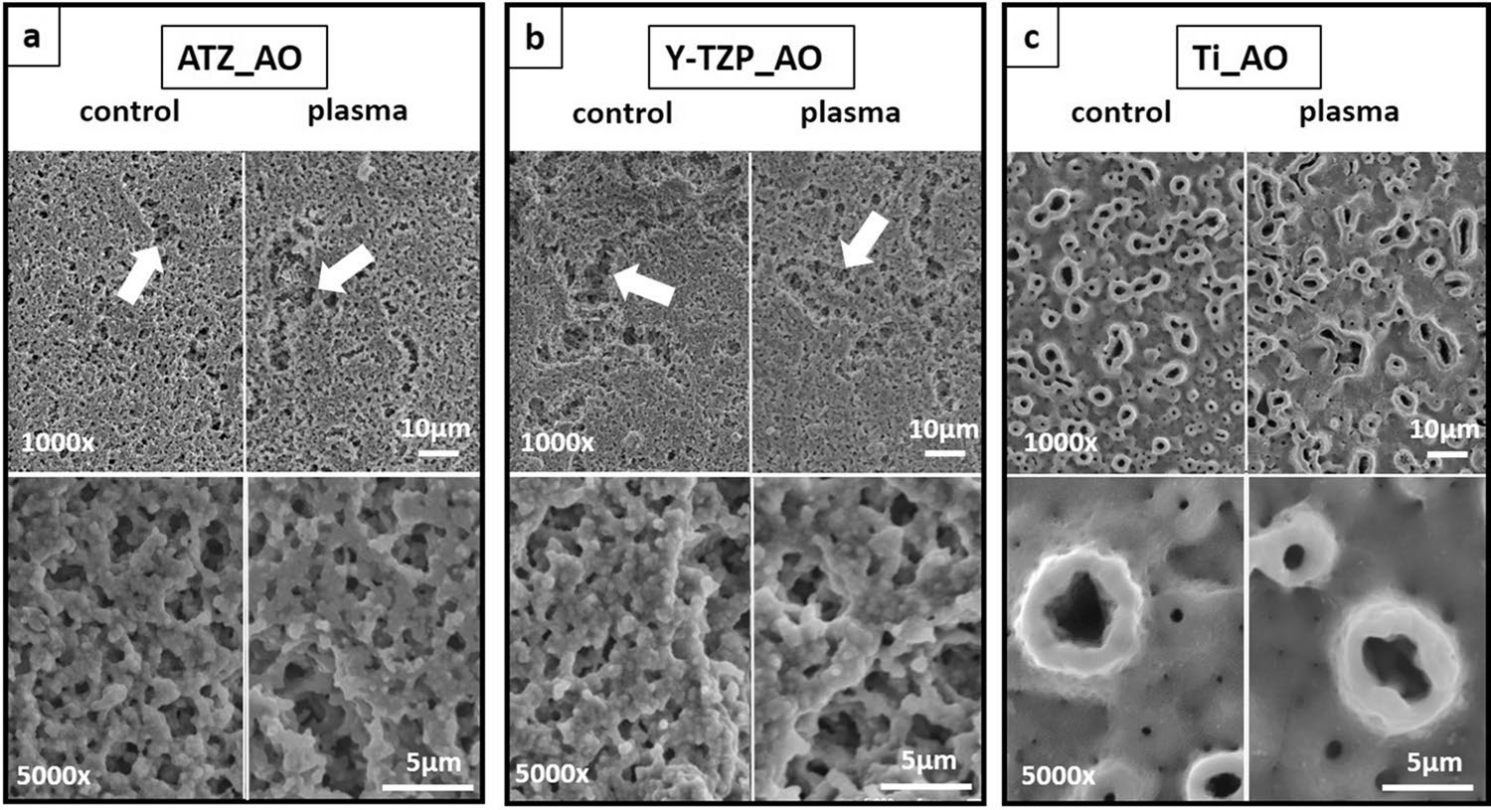
SEM images of ATZ (**a**), Y-TZP (**b**) and titanium (**c**) surfaces for osteoblast culture. Magnifications were set to 1000x (first row) and 5000x (second row). Arrows point to macroscopic grooves and gaps on ceramic surfaces.

As the manufacturing processes of the investigated biomaterial surfaces followed different protocols, we characterized the resulting topographies of the biomaterials by SEM and IFM. Regarding the roughened surfaces for AO culture, SEM images revealed that the microporous ATZ- and Y-TZP-based ZircaPore surfaces differed significantly from the electrochemically anodized titanium discs. The zirconia coating of the ATZ and Y-TZP biomaterials had interconnected micropores with intermittent macroscopic grooves and gaps (Fig. 1a,b; arrows) and displayed a grainy structure at higher magnification (Fig. 1a,b lower row). In contrast, the titanium surfaces (Fig. 1c) had small pores with a diameter of approx. 3–10 µm which were irregularly distributed across the surface. The surface of the pore edges and the spaces in between the pores appeared thereby as smooth surface at the low micrometre level. No differences in surface topography between plasma-functionalized and control surfaces were detected by SEM analysis.

**Figure 2:**
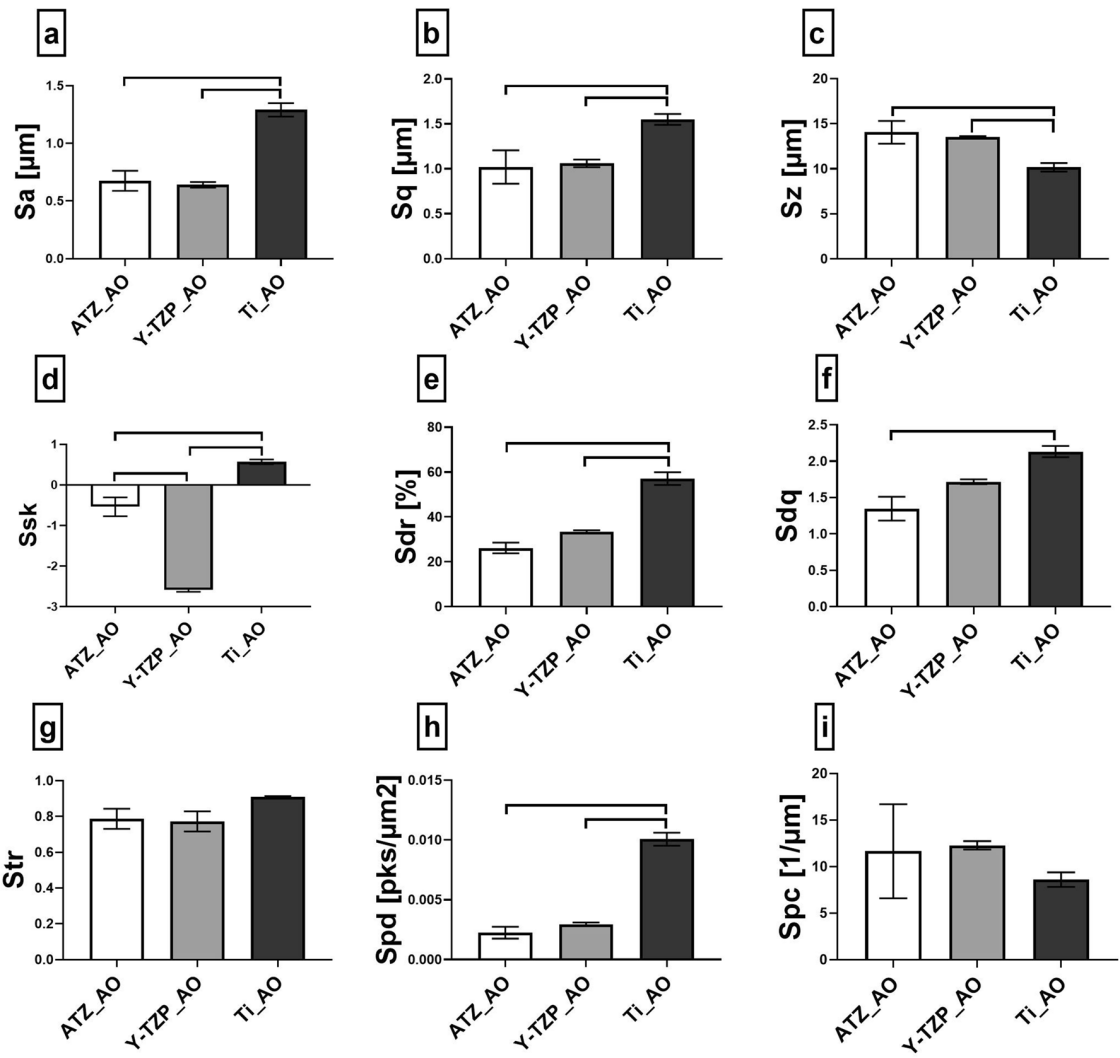
Three-dimensional topographical characterization of osteoblast surfaces by interferometry. Surface parameters describing the topography of biomaterial surfaces were (**a**) S_a_ (average surface height deviation amplitude), (**b**) S_q_ (root-mean-square deviation), (**c**) S_z_ (ten-point height of surface topography), (**d**) S_sk_ (skewness), (**e**) S_dr_ (surface enlargement compared to a totally flat reference area), (**f**) S_dq_ (root mean square gradient, existence of surface slopes), (**g**) S_tr_ (texture aspect ratio), (**h**) S_pd_ (density of peaks) and (**i**) S_pc_ (curvature of peaks). Plots show mean values (n = 4) ± SEM. Statistically significant differences (*p* < 0.05, Tukey´s HSD test) were marked with brackets above the corresponding bars.

Since control and plasma-functionalized surfaces did not differ in their surface topography, the IFM analysis was performed with untreated biomaterials to quantify the differences in surface topography between the three different biomaterials. The IFM analysis presented in Fig. 2 and the Supplementary Table S1 online revealed that the most pronounced differences between zirconia- and titanium-based biomaterials concerned (i) the surface roughness parameters S_a_ and S_q_ (Fig. 2a,b), (ii) the skewness of the surface height distributions S_sk_ (Fig. 2d), (iii) the surface enlargement S_dr_ (Fig. 2e) and (iv) the density of peaks S_pd_ (Fig. 2h). ATZ and Y-TZP yielded comparable values for the surface roughness (S_a_ = 0.68 µm for ATZ and 0.64 µm for Y-TZP, S_q_ = 1.02 µm for ATZ and 1.06 µm for Y-TZP), surface enlargement (S_dr_ = 26.17% for ATZ, S_dr_ = 33.44% for Y-TZP) and peak density (S_pd_ = 0.002 for ATZ, S_pd_ = 0.003 for Y-TZP). Negative values for S_sk_ were found for both ceramic surfaces (S_sk_ = − 0.54 for ATZ and − 2.6 for Y-TZP) meaning that the surface height distribution was shifted towards a predominance of valleys. The titanium surfaces showed, for all aforementioned parameters, statistically significant higher values when compared to ATZ and Y-TZP (S_a_ = 1.3 µm, S_q_ = 1.55 µm, S_sk_ = 0.57, S_dr_ = 57.14%, S_pd_ = 0.01 for titanium surfaces). Further, the number of peaks as measured by S_dq_ was increased on titanium surfaces when compared to the ceramic surfaces (S_dq_ = 2.13 for Ti, 1.35 for ATZ and 1.72 for Y-TZP). Only the comparison of the S_dq_-values from titanium- and ATZ-surfaces reached statistical significance. These data indicate that the anodized Ti surfaces had the roughest surface properties among the biomaterials under study. This, together with a high peak density led to the highest surface enlargement measured for the AO surfaces. Due to the protruding nature of the pores emerging from the titanium surfaces, height distribution was skewed above the mean plane as reflected by the positive value for S_sk_. Regarding the spatial orientation of the surface structures, all examined surfaces had S_tr_ values (Fig. 2g) between 0.77 and 0.91 and therefore did not show a preferred direction of the surface structure.

Since the SEM analysis pointed to smooth surface characteristics between the pores on titanium discs, the higher IFM values for surface roughness, enlargement and peak density seemed rather to reflect the structure and distribution of the pores than the surface in between the pores. Hence, according to the IFM data, the anodized Ti surfaces displayed rougher surface characteristics than the microporous zirconia discs, even though qualitative SEM analysis suggests an inverse trend.

#### Topography of machined surfaces for gingival fibroblasts

**Figure 3:**
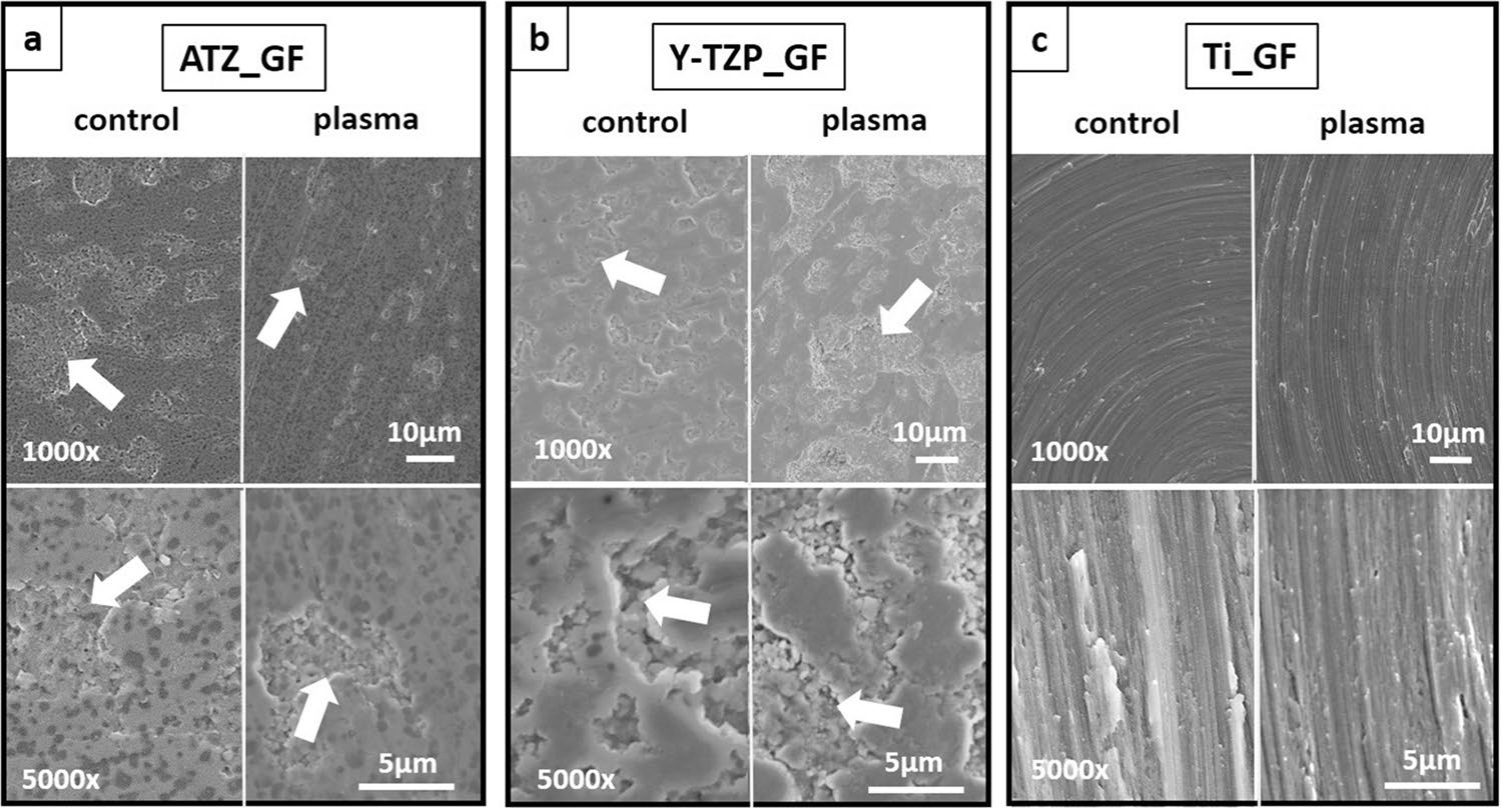
SEM images of ATZ (**a**), Y-TZP (**b**) and titanium (**c**) surfaces for fibroblast culture. Magnifications were set to 1000x (first row) and 5000x (second row). Arrows point to surface depressions on ceramic surfaces.

**Figure 4:**
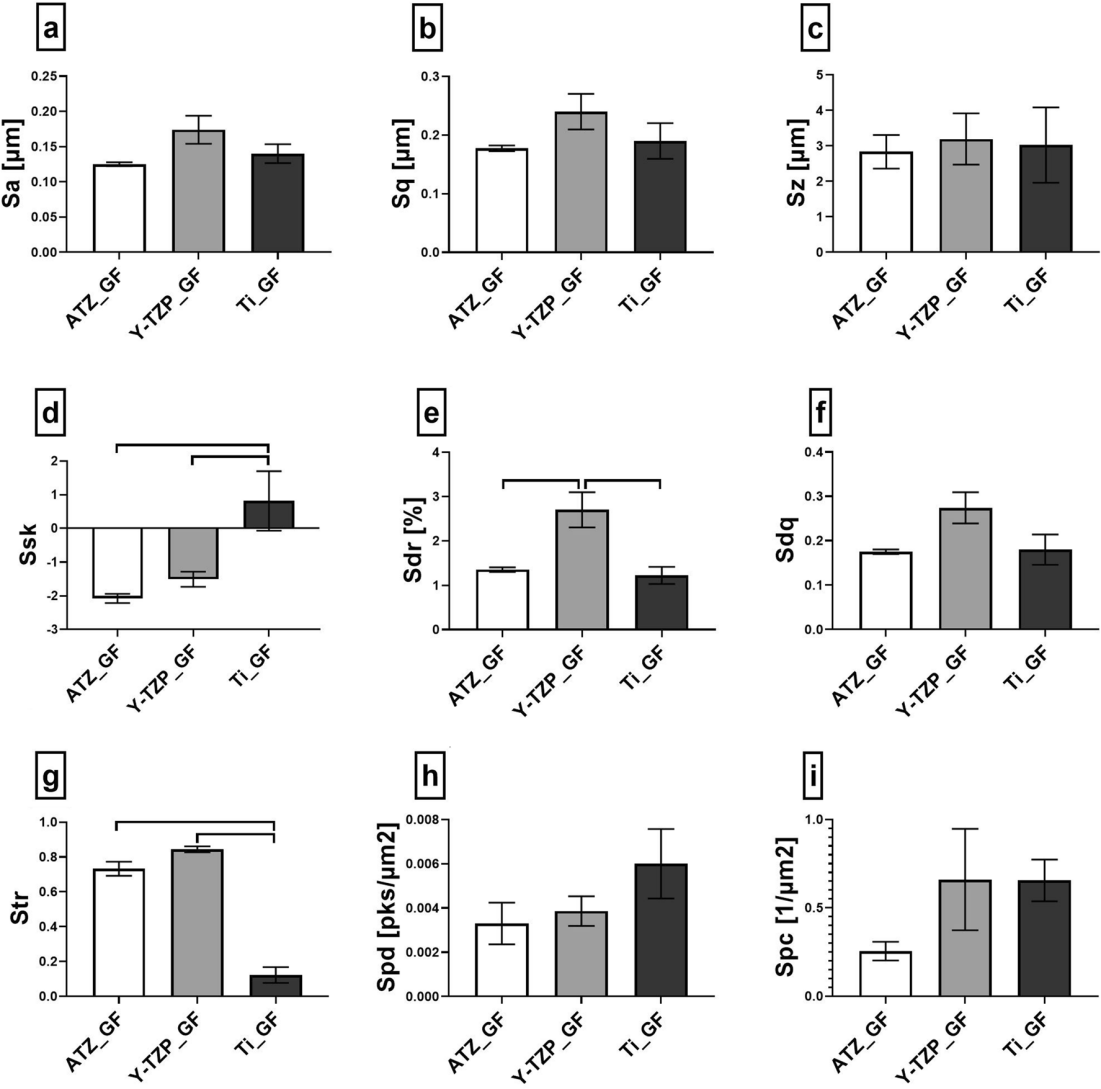
Three-dimensional topographical characterization of fibroblast surfaces by interferometry. Surface parameters describing the topography of biomaterial surfaces were (**a**) S_a_ (average surface height deviation amplitude), (**b**) S_q_ (root-mean-square deviation), (**c**) S_z_ (ten-point height of surface topography), (**d**) S_sk_ (skewness), (**e**) S_dr_ (surface enlargement compared to a totally flat reference area), (**f**) S_dq_ (root mean square gradient, existence of surface slopes), (**g**) S_tr_ (texture aspect ratio), (**h**) S_pd_ (density of peaks) and (**i**) S_pc_ (curvature of peaks). Plots show mean values (n = 4) ± SEM. Statistically significant differences (*p* < 0.05, Tukey´s HSD test) were marked with brackets above the corresponding bars.

Regarding the surfaces for GF culture, ATZ and Y-TZP displayed a plane surface layer that was interrupted by surface depressions in which a microporous surface texture characterized by small hemispherical protuberances predominated (Fig. 3a,b, arrows). On Y-TZP, the plane surface layer appeared to be more ramified and offset from the underlying microrough surface texture than on ATZ. In contrast, the Ti surfaces were characterized by circular heights and depressions, which formed uniform microgrooves all over the surface (Fig. 3c). Control and plasma-functionalized ceramic and titanium surfaces did not differ in their surface topography (Fig. 3). The strong directionality of the grooves on titanium surfaces was reflected by the low surface texture aspect ratio S_tr_ (Fig. 4g, Supplementary Table S2 online) which yielded statistically significant lower values for titanium (S_tr_ = 0.12) when compared to ATZ (S_tr_ = 0.73) and Y-TZP (S_tr_ = 0.84). Machined Ti was the only material group with positive skewness values (S_sk_ = 0.82 µm) (Fig. 4d). This means that the height distribution was located above the mean plane as the surface had more peaks than valleys. With respect to the surface enlargement (Fig. 4e), Y-TZP discs had the highest values (S_dr_ = 2.70%), whereas ATZ and Ti yielded comparable values (S_dr_ = 1.36% for ATZ and S_dr_ = 1.23% for Ti). The other surface parameters, namely S_a/q_ (Fig. 4a,b), S_z_ (Fig. 4c), S_dq_ (Fig. 4f), S_pd_ (Fig. 4h) and S_pc_ (Fig. 4i), did not show statistical significant differences. As a result, both ceramic surfaces for GF culture did not show a specific direction of the surface structure whereas the machined Ti discs had pronounced directional surfaces structures. Regarding the ceramic surfaces, Y-TZP was more structured and therefore displayed a rougher surface.

#### Surface wettability of biomaterial surfaces

**Figure 5:**
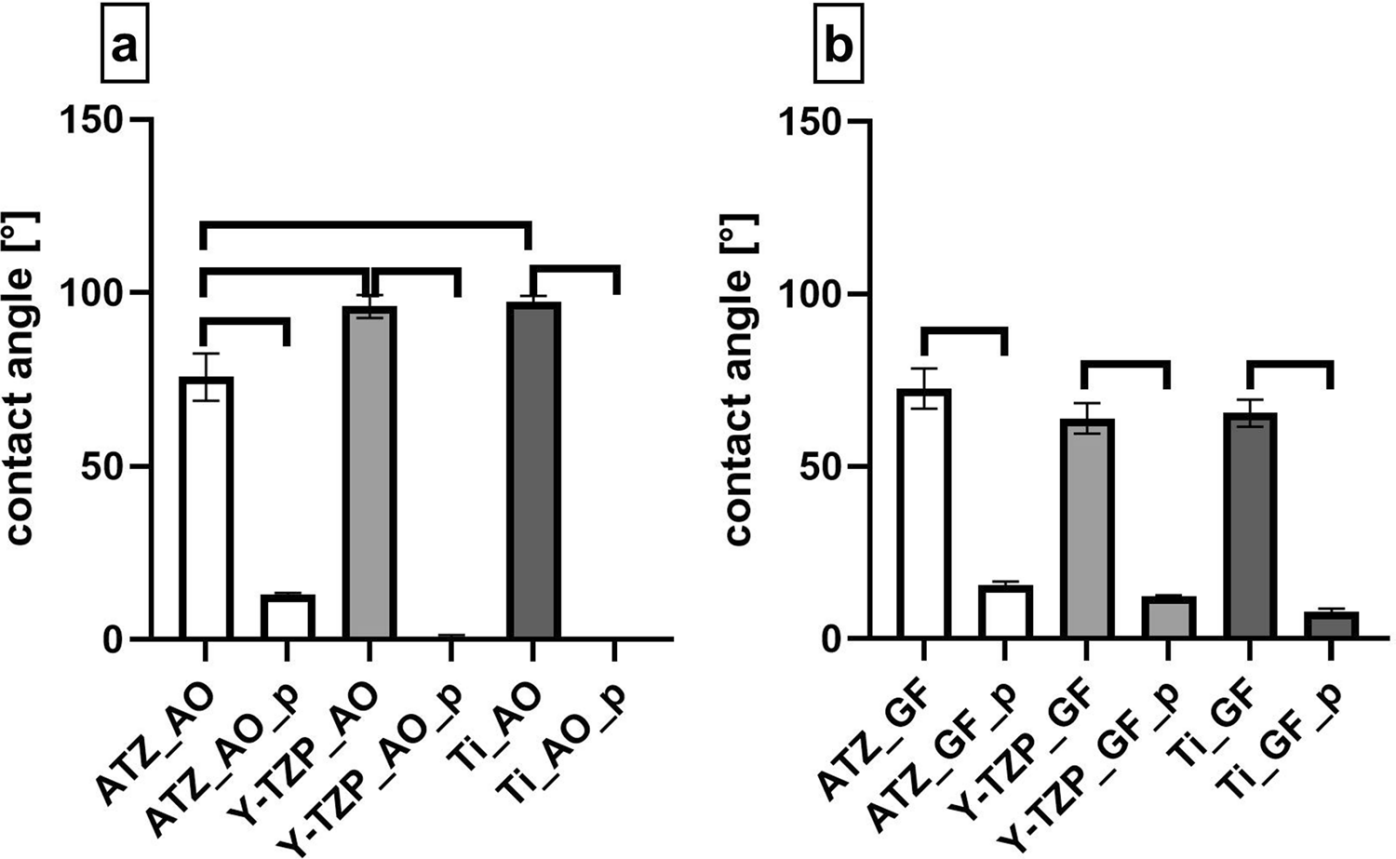
Characterization of biomaterial surface wettability by contact angle measurement. (**a**) Biomaterials intended for osteoblast cultures. (**b**) Biomaterials intended for fibroblast cultures. Graphs show mean values (n = 8) ± SEM. Statistically significant differences (*p* < 0.05, Tukey´s HSD test) were marked with brackets above the corresponding bars.

Surface wettability of the untreated and functionalized biomaterials was analysed by measuring the contact angles of a water droplet on the disc surface. Regarding AO surfaces, contact angles of untreated surfaces were 75.65° for ATZ_AO, 96.04° for Y-TZP_AO and 97.43° for Ti _AO (Fig. 5a, Supplementary Table S3 online). As the contact angle of ATZ_AO was below 90° this biomaterial group can be classified as hydrophilic^44^. The plasma-functionalization resulted in a statistically significant contact angle decrease to 12.90° for ATZ_AO_p (83% decrease), to 0.78° for Y-TZP_AO_p (99% decrease) and to 0.00° for Ti_AO_p (100% decrease). Interestingly, the plasma-based surface hydrophilization was less pronounced on ATZ AO when compared with Y-TZP AO and Ti AO. With respect to the smooth GF surfaces, contact angles of untreated biomaterials were 72.51° for ATZ_GF, 63.86° for Y-TZP_GF and 65.43° for Ti_GF (Fig. 5b, Supplementary Table S3 online). When comparing the contact angles of Y-TZP_GF and Ti_GF with the corresponding osteoblast surfaces Y-TZP_AO and Ti_AO, it is noticeable that the machined biomaterials for GF culture had lower contact angles and therefore showed better wettability than their roughened counterparts. This was however less pronounced for the ATZ-based surfaces. The difference in contact angles between AO and GF surfaces amounted to 3.1° for ATZ (compare Fig. 5a,b), whereas the contact angles of roughened and smooth Y-TZP and Ti surfaces differed of about 32° (compare Fig. 5a,b). After plasma-functionalization, the values for the contact angles of GF surfaces decreased to 15.71° for ATZ_GF_p (77% decrease), to 12.34° for Y-TZP_GF_p (81% decrease) and to 7.94° for Ti_GF_p (88% decrease). Compared to the corresponding roughened surfaces, the contact angle decrease after plasma-functionalization was lower for the smooth biomaterial surfaces. In summary, with exception of the ATZ-based surfaces the untreated roughened biomaterials were hydrophobic whereas the corresponding machined untreated surfaces were hydrophilic. Plasma-functionalization then resulted in an increase of the hydrophilicity of all surfaces, whereas the plasma effect was less pronounced for the smooth GF biomaterials﻿.

In order to grasp the correlation between surface topography and wettability statistically, a Pearson´s Correlation Test was performed. As presented in Table 1, the correlation between contact angles of the control surfaces and S_a_/S_q_, S_dr_ and S_dq_ yielded values for the Pearson´s Correlation Coefficient that suggested a strong correlation between these parameters. The decrease of the contact angles through plasma-functionalization showed also dependency from these topographical parameters. Further, the decrease in contact angle by plasma-functionalization correlated to the initial surface wettability, meaning that the higher the initial contact angle, the stronger was the decrease in contact angle induced by functionalization. This led to an inverse correlation of the contact angles of the functionalized surfaces and S_a_/S_q_, S_dr_ and S_dq_. Therefore, the higher surface roughness, surface enlargement and the more surface elevations existed, the lower was the initial wettability. Surfaces with low wettability then demonstrated the most pronounced effect of plasma-functionalization on surface wettability.

**Table 1:** Correlation between topographical surface parameters (of the control surfaces) and the contact angles (of functionalized and control surfaces).

	Contact angle control surfaces	Contact angle decrease by plasma-functionalization	Contact angle plasma-functionalized surfaces
Sa	0.84*	0.75	− 0.71
Sq	0.88*	0.77	− 0.72
Sz	0.72	0.57	− 0.49
Ssk	− 0.09	0.21	− 0.21
Str	0.48	0.11	− 0.09
Sdr	0.91*	0.81*	− 0.77
Sdq	0.92*	0.81*	− 0.76
Spd	0.37	0.58	− 0.61
Spc	0.76	0.62	− 0.55
Contact angle control surfaces		0.85*	− 0.82*

The table lists the values obtained for the Pearson´s Correlation Coefficient. Statistically significant correlations (two-tailed t-test, *p* < 0.05) are marked with asterisks. The Pearson´s test for correlation provides values ranging from − 1 to 1, whereby values close to − 1 suggest an inverse correlation, values around 0 reject any correlation and values close to 1 point at strong correlation between two parameters.

### Cell morphology

#### Cell morphology of osteoblasts

**Figure 6:**
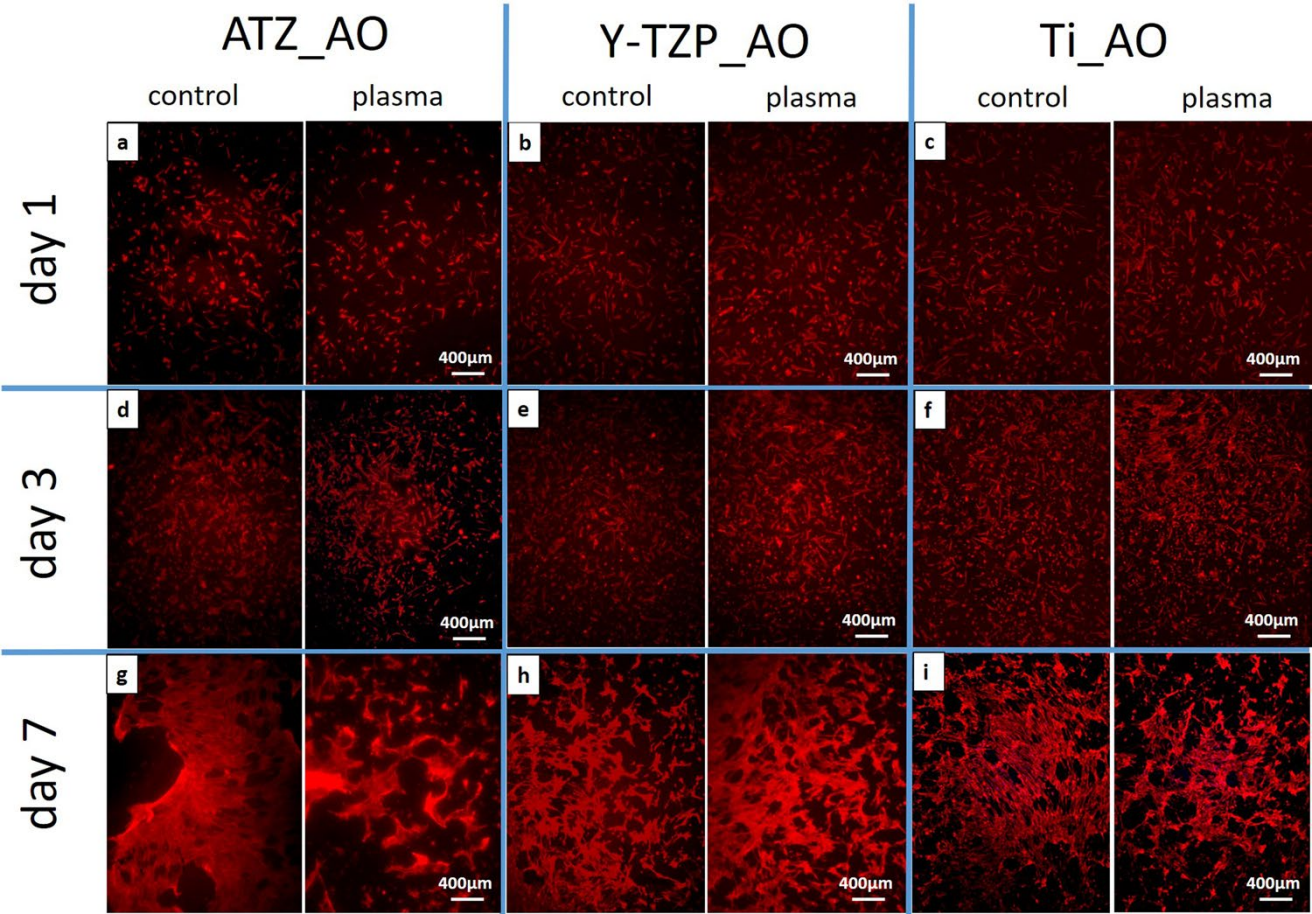
Immunofluorescence of phalloidin-labelled actin (red fluorescence) in osteoblasts cultivated for 1, 3 or 7 days on plasma-functionalized and control surfaces.

**Figure 7:**
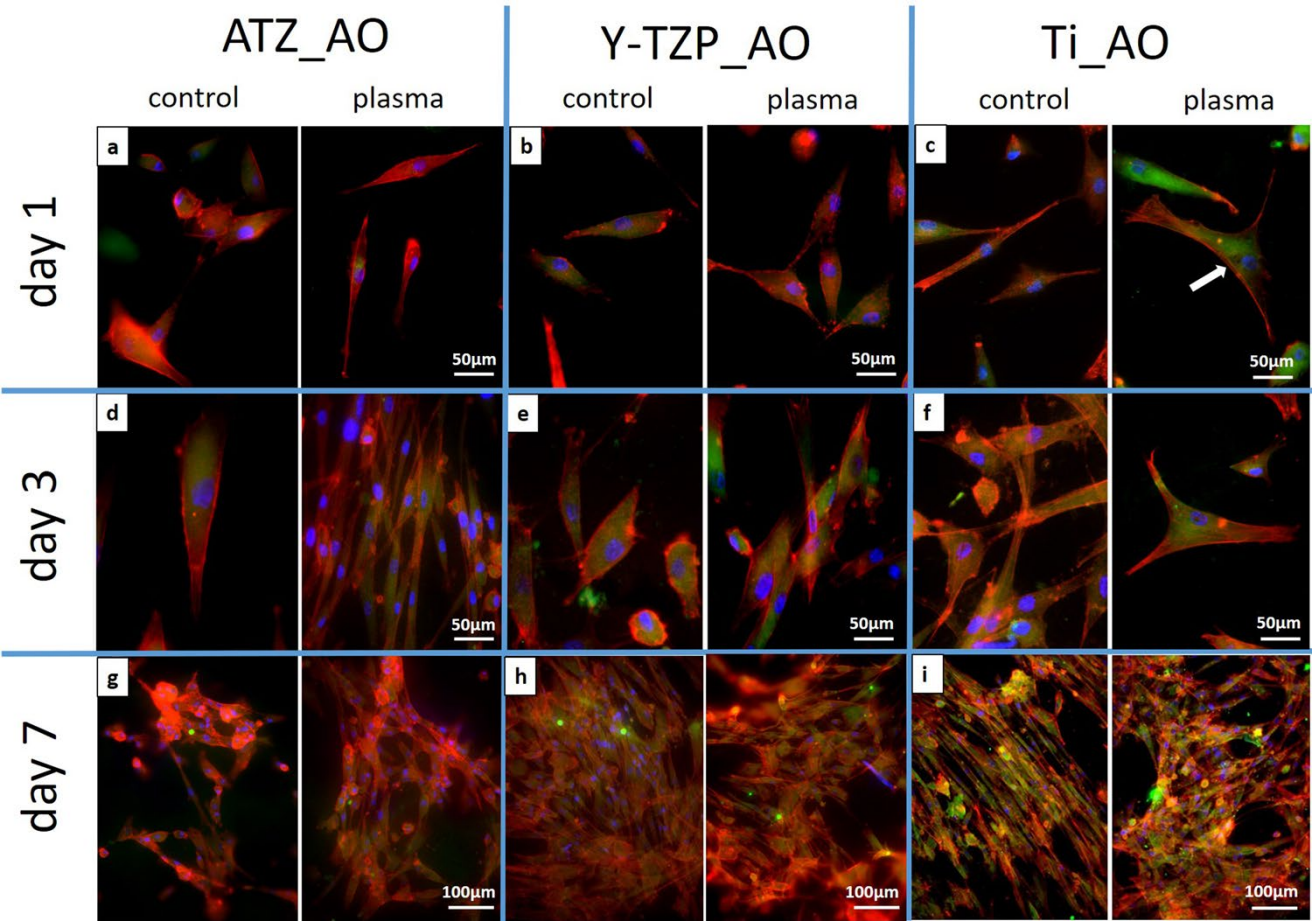
Indirect immunofluorescence of the phalloidin-labelled actin (red fluorescence) and the focal adhesion protein vinculin (green fluorescence) in osteoblasts cultivated for 1, 3 or 7 days on plasma-functionalized and control surfaces. Nuclei were counter-stained with Hoechst 33342 (blue fluorescence). Arrows point to actin stress fibres.

Fluorescence microscopy of actin and vinculin was used to examine 1) the density of the cells on the biomaterial surfaces at days 1, 3 and 7 of cell culture, 2) the organization of the actin cytoskeleton and the focal adhesion protein vinculin at days 1, 3 and 7 of cell culture, and 3) the cell shape/morphology by quantitative morphometry at days 1 and 3. Images of fluorescence-based staining of the actin cytoskeleton (red fluorescence) for the analysis of cell density are shown in Fig. 6. Micrographs for the qualitative analysis of the actin cytoskeleton and the distribution of vinculin (green fluorescence) are depicted in Fig. 7 (left images: control surfaces, right images: plasma-treated surfaces). Plasma-functionalized surfaces showed a comparable cell density as the untreated controls at all investigated time points (Fig. 6a–i). The cell layer on functionalized and non-functionalized ATZ-surfaces (Fig. 6g) was partly detached at day 7 suggesting weaker cell adhesion to the ATZ-surfaces compared to Y-TZP- and Ti-surfaces. Regarding the organization of actin and vinculin (Fig. 7), no considerable differences between plasma-functionalized and untreated surfaces could be revealed by qualitative assessment of the micrographs. Differences in cell morphology, namely actin organization and temporal distribution of vinculin, were however detectable between the biomaterial groups. In detail, on ATZ_AO (Fig. 7a) and Y-TZP_AO (Fig. 7b) actin was diffusely distributed in the cell soma and formed dot-shaped signals at the cell margins at day 1 of culture. In contrast, cells on corresponding Ti-based surfaces were more spread and showed stress fibre formation at high cell tension sites at the cell margins and at large attachments sites (Fig. 7c arrows). The fluorescence intensity of vinculin signals appeared to be less intense on ceramic than on Ti surfaces (compare Fig. 7a,b with Fig. 7c). At day 3, the actin cytoskeleton in AO on ceramic materials adopted similar organization as observed on Ti (compare Fig. 7d,e with Fig. 7). With respect to the vinculin distribution in AO, the qualitative microscopic analysis revealed an increase in vinculin intensity on ceramic surfaces at day 3, reaching thereby comparable patterns with matched Ti-based surfaces (Fig. 7d–f). This cytoskeletal organization persisted on ceramic biomaterials until day 7 (Fig. 7g–h), whereas on Ti-based surfaces the vinculin fluorescence appeared to be more pronounced than in cells on ATZ and Y-TZP (compare Fig. 7g,h with Fig. 7i). These results point to a biomaterial- and time-dependent organization of the cytoskeleton, which was characterized by a less pronounced actin stress fibre formation and delayed vinculin expression in AO on ceramic surfaces when compared with cells on Ti.

**Figure 8:**
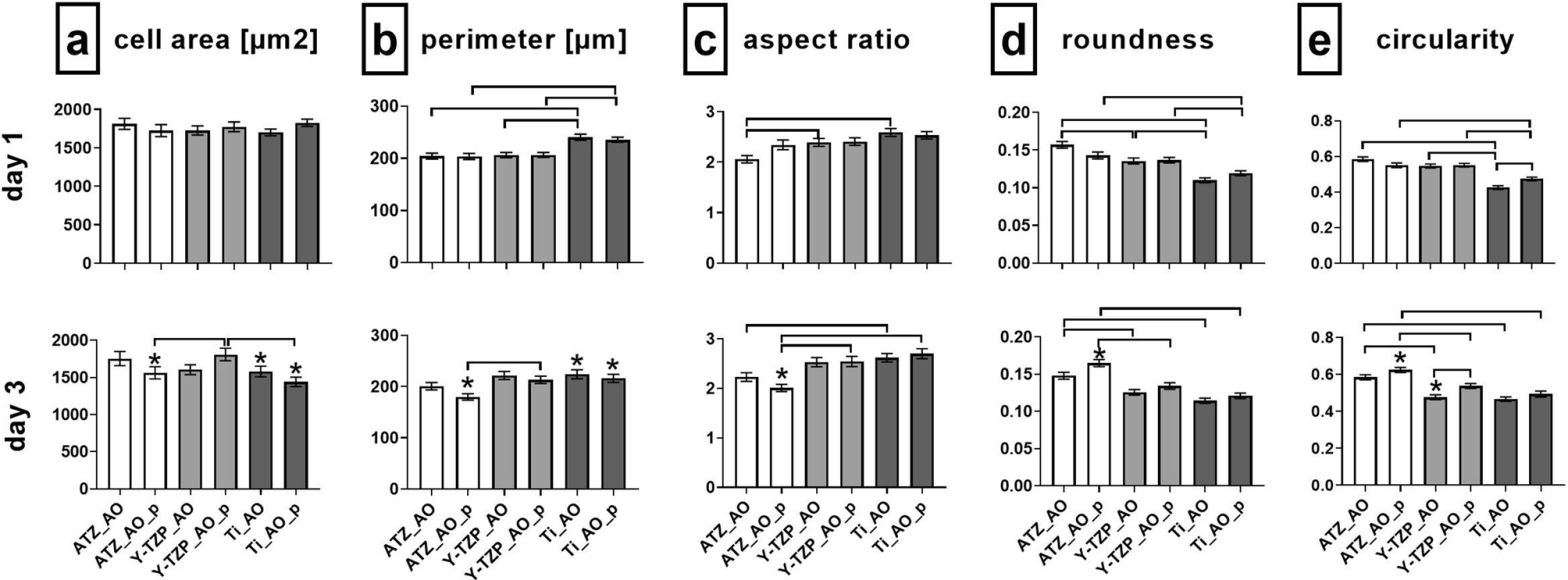
Quantitative morphometric analysis of osteoblast morphology after 1 and 3 days of culture on untreated and plasma-functionalized surfaces. Since the cells had reached confluence at day 7, no morphometric analysis was possible at day 7. Data are presented as mean values (194 < n < 373) ± SEM. Statistically significant differences (*p* < 0.05, Dunn´s HSD test) were marked with brackets above the corresponding bars. “*” marks statistically significant differences between day 1 and 3.

The quantitative morphometric analysis (Fig. 8, Supplementary Table S4 online) demonstrated that the only morphometric parameter differing statistically significant between AO on untreated and plasma-functionalized surfaces was cell circularity on Y-TZP- and Ti-based biomaterials (Fig. 8e). As reflected by higher values for circularity on Ti_AO_p versus untreated Ti_AO at day 1 (*p* = 0.0295) and on Y-TZP_AO_p versus Y-TZP_AO at day 3 (*p* = 0.0451), AO on plasma-functionalized surfaces seemed to form less cellular protrusions when compared to cells on corresponding untreated surfaces. When comparing the AO morphology in terms of cell shape between the three material test groups, the morphometry data at day 1 further revealed higher cell perimeter (Fig. 8b), lower roundness (Fig. 8d) and circularity (Fig. 8e) on Ti-based surfaces than on ATZ- and Y-TZP- based surfaces. AO were, hence, more spread and formed more cellular protrusions on Ti than on the ceramic-based materials. The morphological difference was still present between Ti- and ATZ-based surfaces after 3 days of culture. This observation is in line with the lower aspect ratio (Fig. 8c) and higher roundness (Fig. 8d) values of AO on ATZ-based surfaces versus Y-TZP and Ti at day 1, and in part at day 3, suggesting the least spread cell morphology on ATZ. Regarding the temporal changes in cell morphology, the results demonstrated a decrease in cell area (Fig. 8a) and perimeter (Fig. 8b) on Ti-based surfaces at day 3, indicating a morphological reorganization of the cells from day 1 to day 3 towards a more compact cell shape. A similar cell response was observed on plasma-functionalized ATZ_AO_p surfaces. AO morphology at day 3 was characterized by a lower cell area, perimeter and aspect ratio which coincided with higher values for roundness and circularity when compared to values at day 1. Altogether, these data indicate the formation of a more rounded cell shape with less cellular protrusions on ATZ_AO_p at day 3.

In summary, the qualitative (Fig. 7) and quantitative (Fig. 8) analysis of AO morphogenesis demonstrated that the effect of plasma-based biomaterial functionalization was weak. AO morphogenesis including cytoskeletal organization and cell shape was rather modulated in a biomaterial- and time-dependent manner.

#### Cell morphology of gingival fibroblasts

**Figure 9:**
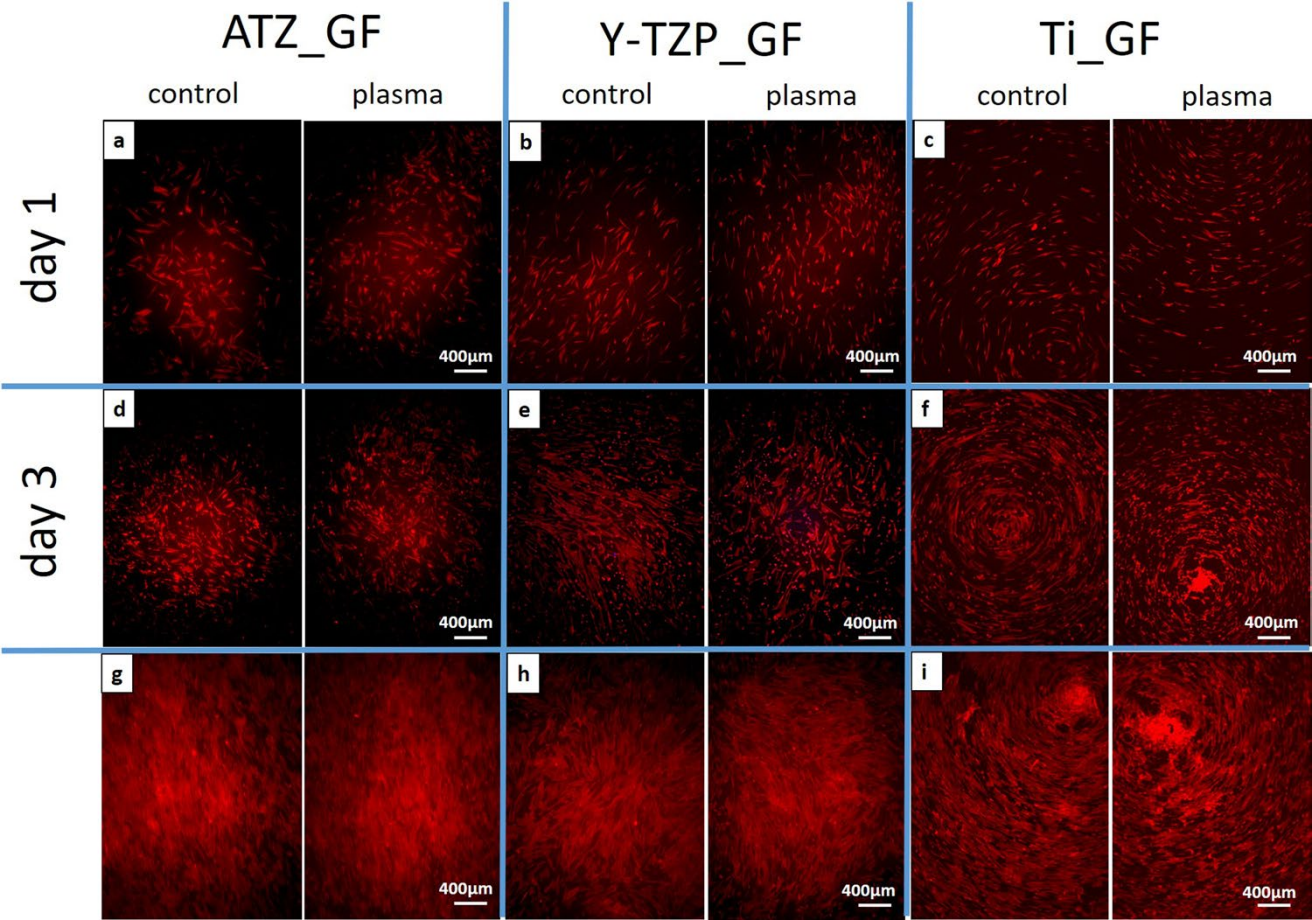
Immunofluorescence of phalloidin-labelled actin (red fluorescence) in fibroblasts cultivated for 1, 3 or 7 days on plasma-functionalized and control surfaces.

**Figure 10:**
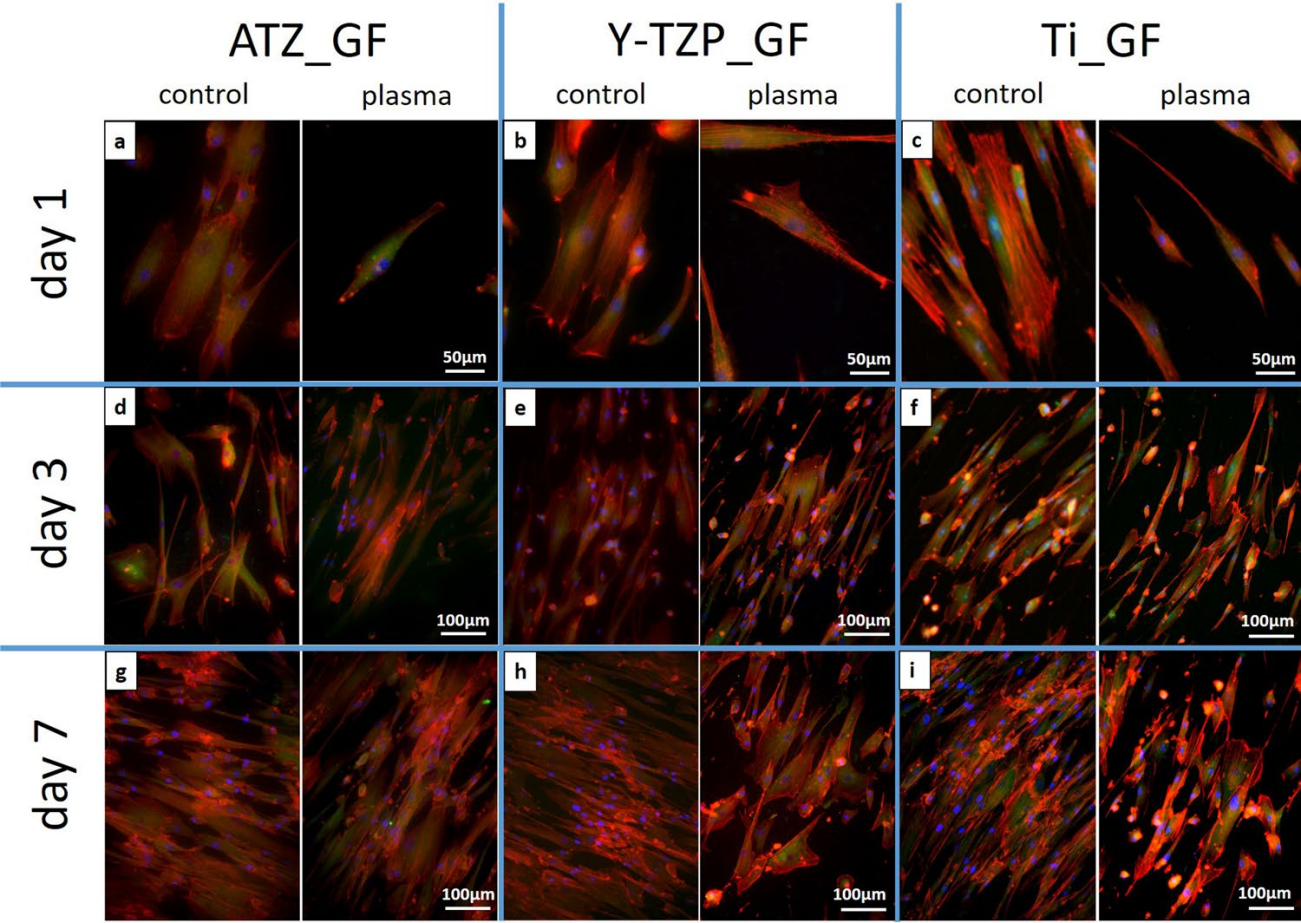
Indirect immunofluorescence of the phalloidin-labelled actin (red fluorescence) and the focal adhesion protein vinculin (green fluorescence) in fibroblasts cultivated for 1, 3 or 7 days on plasma-functionalized (large images) and control surfaces (inserts). Nuclei were counter-stained with Hoechst 33342 (blue fluorescence).

The cell density and the morphogenesis of GF on the test biomaterials was examined in analogy to AO cells. Plasma-functionalized and untreated control surfaces demonstrated a comparable cell density for all biomaterials at all time points under study (Fig. 9a–i). The grooved titanium surfaces favoured cell elongation alongside the grooves (Fig. 9c,f,i). Further, the qualitative analysis of the actin cytoskeleton and the focal adhesion protein vinculin revealed no difference in actin organization and vinculin intensity or distribution between plasma-treated and untreated materials (Fig. 10; left images: control surfaces, right images: plasma-treated surfaces). In general, GF were spread and elongated on all biomaterials, with pronounced actin stress fibre formation and detectable vinculin fluorescence at all culture time points under study. This observation points to a fast and stable adhesion of GF on the smooth surfaces.

**Figure 11:**
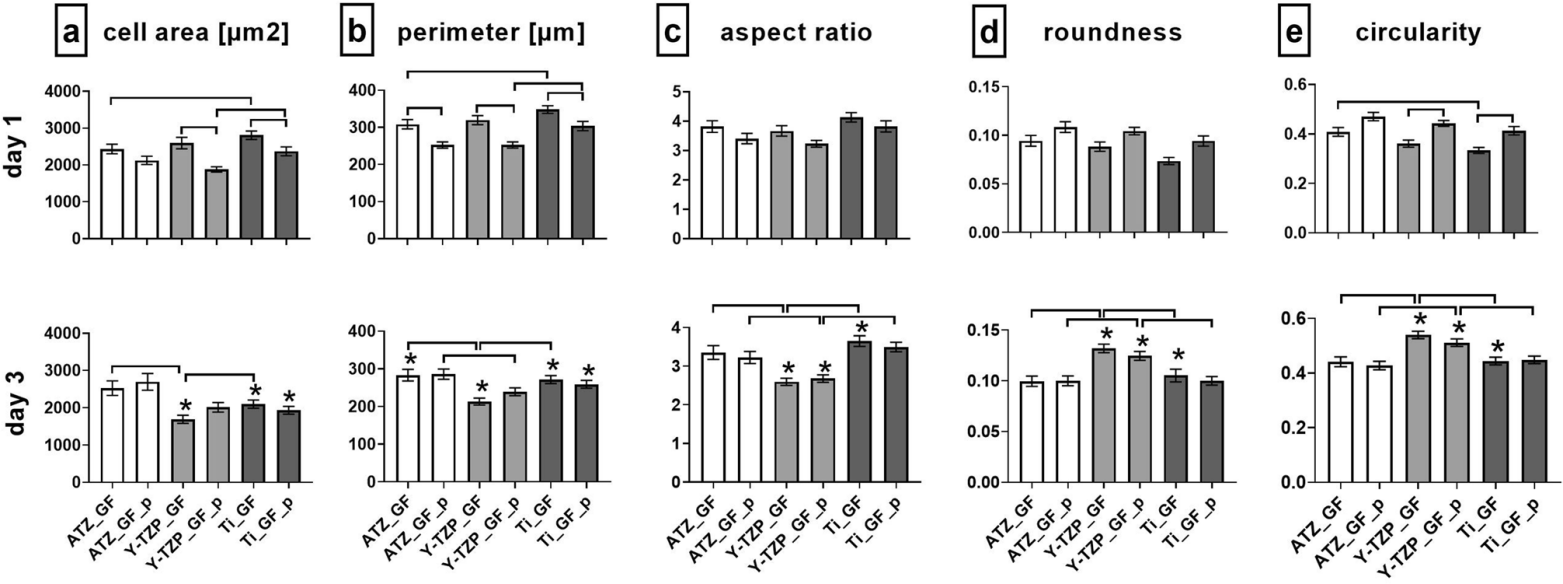
Quantitative morphometric analysis of fibroblast morphology after 1 and 3 days of culture on functionalized and control surfaces. Since the cells had reached confluence at day 7, no morphometric analysis was possible at day 7. Data are presented as mean values (145 < n < 300) ± SEM. Statistically significant differences (*p* < 0.05, Dunn´s HSD test) were marked with brackets above the corresponding bars. “*” marks statistically significant differences between day 1 and 3.

The evaluation of GF morphogenesis in terms of cell shape revealed that plasma-functionalization of the surfaces induced distinct cell morphologies when compared with untreated materials at day 1 (Fig. 11, Supplementary Table S5 online). The morphometry data thereby revealed a less spread morphology with fewer cellular protrusions on plasma-treated surfaces than on the corresponding untreated materials. The morphological differences were indicated by lower values for cell area (Fig. 11a; ATZ: *p* = 0.6416; Y-TZP: *p* < 0.0001, Ti: *p* = 0.0088), cell perimeter (Fig. 11b; ATZ: *p* = 0.0289, Y-TZP: *p* < 0.0001, Ti: *p* = 0.0008) and aspect ratio (Fig. 11c; ATZ: *p* = 1.0, Y-TZP: *p* = 0.3825, Ti: *p* = 0.1465), and higher roundness (Fig. 11d; ATZ: *p* = 0.4477, Y-TZP: *p* = 0.1471, Ti: *p* = 0.2163) and circularity (Fig. 11e; ATZ: *p* = 0.1128, Y-TZP: *p* = 0.0004, Ti: *p* = 0.1322) for the plasma-treated samples. The different emerging cell morphologies are depicted in Fig. 10a–c (compare left with right images). At day 3, however, the effect of plasma-functionalization on cell morphogenesis was no longer evident (Fig. 11, lower panel). GF reorganized, with the exception of cells on ATZ, towards a more rounded morphology with less cellular protrusions. This is reflected by decreased values of cell area, perimeter and aspect ratio, as well as higher roundness and circularity (Fig. 11, lower panel). Such a cellular reorganization towards a more compact morphology from day 1 to day 3 has also been observed for AO on Ti_AO and ATZ_AO_p.

### Metabolic activity and proliferation

#### Metabolic activity and proliferation of osteoblasts

**Figure 12:**
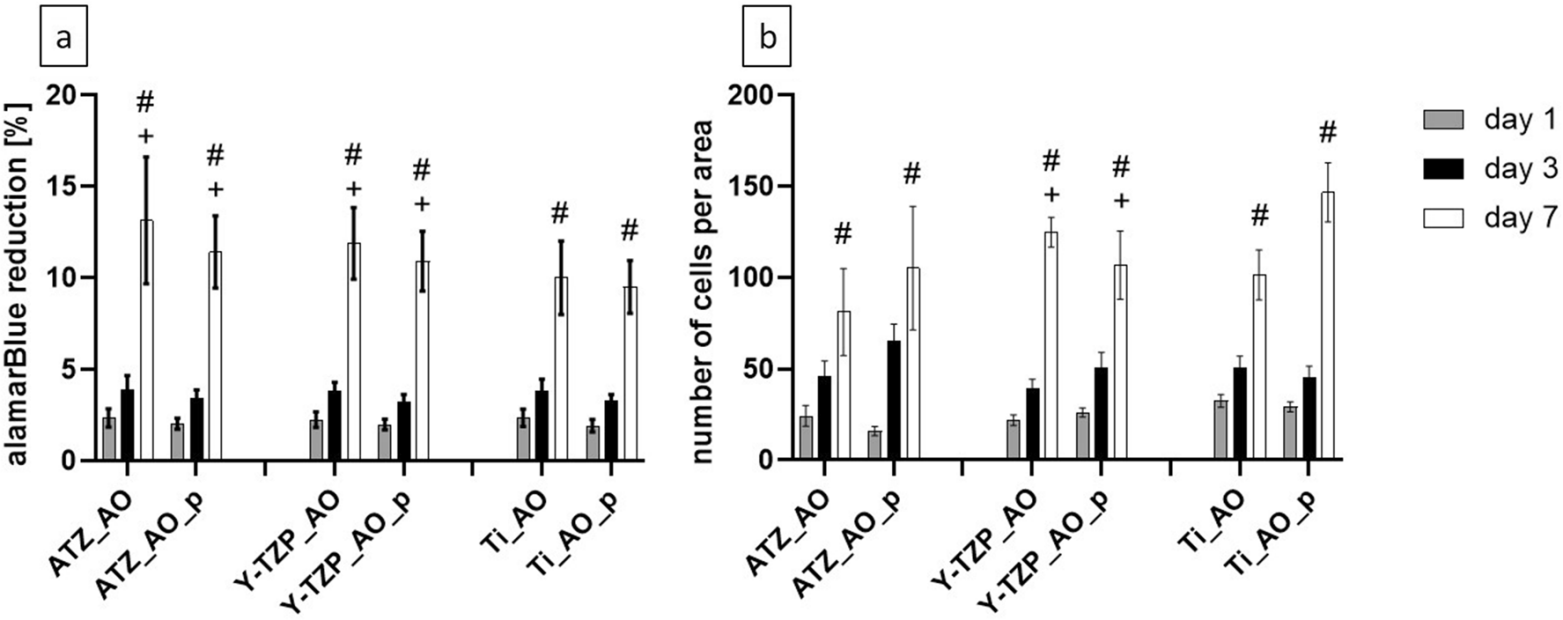
Metabolic activity (**a**) and total cell number per area determined by counting the cells on micrographs (**b**). The percentage of alamarBlue reduction in the supernatant refers to a 100% reduced control. Data are presented as mean values ± SEM (data collected on 6 biomaterial discs). No statistically significant differences were found between the different biomaterial groups. “+” marks statistically significant differences between day 3 and day 7 and “#” between day 1 and day 7.

Since we observed a biomaterial- and time-associated modulation of AO morphogenesis on the different biomaterials, we next examined the metabolic activity and proliferation of AO at days 1, 3 and 7. Regarding the metabolic activity of AO (Fig. 12a, Supplementary Table S6 online) and the number of attached cells (Fig. 12b, Supplementary Table S6 online) on the different surfaces, our data demonstrate a continuous increase in cell metabolic activity and proliferation from day 1 to day 7 on all biomaterials under study, irrespective of plasma-functionalization or type of biomaterial. The alamarBlue reduction and the number of attached cells were not significantly different between cells on plasma-functionalized and control surfaces. This result suggests that plasma-functionalization did not alter the metabolic activity and proliferation of AO.

#### Metabolic activity and proliferation of gingival fibroblasts

**Figure 13:**
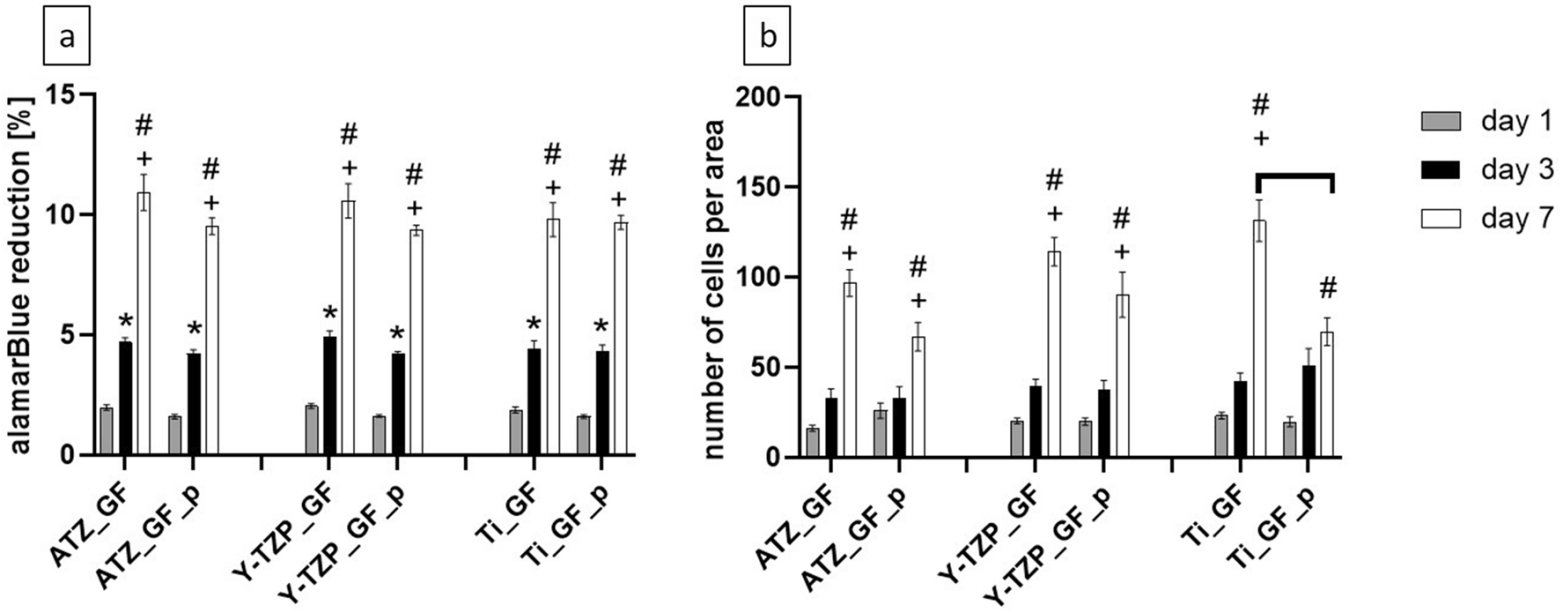
Metabolic activity (**a**) and total cell number per area determined by counting the cells on micrographs (**b**). The percentage of alamarBlue reduction in the supernatant refers to a 100% reduced control. Data are presented as mean values ± SEM (data collected on 6 biomaterial discs). Statistically significant differences between the different biomaterial groups were marked with brackets above the corresponding bars. “*” marks statistically significant differences between day 1 and 3, “+” between day 3 and day 7 and “#” between day 1 and day 7.

With respect to the metabolic activity of GF on the test biomaterials, we could not demonstrate a significant difference between the alamarBlue reduction on plasma-functionalized and control surfaces (Fig. 13a, Supplementary Table S7 online). On all biomaterials the alamarBlue reduction increased significantly from day 1 to day 7, thus indicating a constant proliferation up to day 7. This observation was further confirmed by the determination of the cell number (Fig. 13b, Supplementary Table S7 online). It is however of note, that the counted cell number on untreated Ti_GF was significantly higher than on plasma-treated Ti_GF_p at day 7 (Fig. 13b). These results suggest that plasma-functionalization had a weak effect on initial GF morphology whereas the metabolic activity and proliferation of GF appeared to be not affected by plasma-functionalization.

## Discussion

Several studies have demonstrated that the functionalization of oral implants and their suprastructures with gas plasmas can create favourable conditions for the integration of the implants into the target tissue^9,13,15–18,20–24,26,29,31,45,46^. Against this background, the authors firstly examined the response of AO and GF to three clinically established oral implant surfaces with different topographies and functionalized with oxygen plasma over a period of 7 days. Oxygen plasma was generated using a commercially available plasma-cleaner, which would allow for the chairside functionalization of oral implants and their suprastructures directly prior to insertion.

In order to describe the cellular response as a function of the surface properties, we first characterized the surface topography and wettability of the biomaterials. This characterization demonstrated that the biomaterial surfaces for AO and GF did not only differ in topographical surface aspects but also showed differences in their wetting behaviour. Non-functionalized rough Y-TZP and titanium surfaces for AO were hydrophobic^44^, whereas corresponding smooth surfaces for GF had hydrophilic characteristics. This observation indicates that the surface topography of the examined biomaterials played a decisive role for their surface wetting behaviour. The statistical correlation analysis of the surface topography parameters and the contact angles confirmed this assumption and revealed that a high surface roughness, surface enlargement and many surface elevations yield a lower initial wettability. This is in line with other reports ascribing initial hydrophobicity of implant surfaces to surface roughness^47,48^. The hydrophobic surface features of roughened surfaces have been explained by the Cassie-Baxter wetting state. This theory describes the entrapment of air in surface pores as a cause for the formation of a heterogenous solid/air surface that impairs the spontaneous wetting of the surface^48–50^. However, the wettability of “rough” and “smooth” ATZ-surfaces in the present investigation was similar, suggesting that in this case the chemical surface composition of the ATZ discs had a stronger effect on the surface wettability than the structural surface features.

With regard to the effect of plasma-functionalization on biomaterial surface characteristics, we found—in accordance with other studies—that plasma-functionalization did not alter the surface topography^18,23,31,51,52^, but significantly increased the surface wettability of the implant surfaces. This finding is in line with former investigations^15,18,21,26,53–55^, which ascribed the increase in wettability to a removal of surface carbon contaminations by the plasma-functionalization process^11,13,15,23,27,30,51,52,54^. Interestingly, our data demonstrated that the plasma-induced increase of the surface wettability was generally lower for the smooth GF/abutment surfaces. Overall, these results indicate that the plasma-induced increase in surface wettability depended on specific surface parameters. More precisely, surface wettability was related to the surface roughness, and on the initial wettability of the materials prior to functionalization. This observation is corroborated by Noro et al.^54^ and Koban et al.^56^ who also reported a less pronounced increase in hydrophilicity on smooth surfaces following plasma-functionalization. The present results, therefore, suggest that the level of hydrophilicity obtained by plasma-functionalization depends on the initial biomaterial surface characteristics. A former work by our group presenting an implant topography-dependent effect on the functionalization efficacy of implant surfaces by UV light^37^ further supports this assumption.

Regarding the AO response to plasma-functionalized implant surfaces, our results revealed that cell attachment, morphogenesis, metabolic activity and proliferation were hardly affected when compared to untreated controls. The results of this investigation are in contrast to previous investigations characterizing the response of osteoblast-like cells to implant biomaterials functionalized with oxygen plasma^11,23,27^. Despite the similar experimental set-up with a commercially available plasma-cleaner, the positive effect of oxygen plasma-functionalization on cell attachment and proliferation found in previous investigations^11,23,27^, could not be reproduced in the present investigation. One reason for the deviating results in the present investigation might be the use of biomaterials with different topographies and chemical compositions when compared to the prior investigations. Referring to this, Guo et al.^29^ stated that the effects of plasma-functionalization on the cell response varied with the type of the treated material. Furthermore, the differing results regarding the osteoblast response to plasma-functionalized surfaces might be attributed to the different in vitro cell culture models used in former investigations. The previous studies investigating the effects of oxygen plasma-functionalization on the cellular response were performed with osteoblast-like cells, namely the osteosarcoma cell line MG-63^23^ or with murine osteoblastic cells^11,27^. These cell lines, however, differ in their response to biomaterials from primary human osteoblasts^33,36^. For instance, it has been reported that that the “influence of the topography on human osteoblastic MG-63 cells is reduced by the chemical and physical properties of the surface”^21^. In contrast we found in the present work and in a former investigation^23^ that primary human osteoblasts react more sensitively to alterations in surface topography than to changes in surface wettability. The influence of the surface topography on AO response in the present investigation was characterized by a more pronounced expression of vinculin and actin stress fibre formation as well as a more spread morphology on titanium surfaces when compared to the ceramic surfaces under study. Vinculin is a protein involved in cell adhesion to an extracellular matrix or artificial surface as it couples cytoskeletal elements such as actin fibres with adhesions receptors like integrins thereby stabilizing and promoting the growth of focal adhesions^57^. In the context of cell-biomaterial interaction it has been demonstrated that vinculin is a sensitive marker for cell adhesion, as the degree of vinculin-associated plaque formation correlates with the level of cell attachment^58^. Therefore, the vinculin distribution on the titanium-based surfaces in the present work indicated a faster and/or stronger initial cell attachment compared to the ceramic surfaces under study. Furthermore, AO on Ti formed pronounced actin stress fibres between large adhesion sites. Such a cytoskeletal organisation as well as the observed spread morphology detected by morphometry are usually seen on surfaces that are smooth at the low micrometre level^7,55^. As the surface in between the pores on the titanium biomaterials appeared smooth, the observed osteoblast morphology suggests that electrochemically anodized surfaces represent smooth surfaces seen from the cell´s point of view.

With respect to the GF behaviour on the smooth plasma-functionalized abutment surfaces, we identified a differential morphogenesis on plasma versus control materials. The impact on GF morphology was thereby exclusively detectable at day 1 and characterized by a less spread and more rounded cell shape with a reduced number of cellular protrusions. A reduced cell spreading and formation of cellular protrusions points to a delayed cell adhesion on the plasma-functionalized surfaces^22,59^. However, the pronounced actin stress fibre formation and the presence of vinculin on all smooth biomaterial surfaces at day 1 indicates a stable cell attachment to the biomaterials under study—irrespective of the plasma-treatment. This is in line with our data from the proliferation analysis, which revealed no significant differences between GF on plasma-treated and untreated control surfaces. The present results thus suggest that the effect of plasma-functionalization on the GF response was restricted to morphological changes within the first 24 h of the cell-biomaterial interaction. These findings contradict with the results of Guo et al.^29^ who found increased fibroblast attachment on titanium and zirconia surfaces. The differing results between the investigation of Guo et al.^29^ and the present study might again be explained by the different biomaterial surfaces that were used in the investigations. It is further of note that the study of Guo et al.^29^ was limited to the first 48 h after cell seeding. The results of the present investigation point however to a transient effect of plasma-functionalization on GF behaviour. It therefore remains to be proven whether the previously described beneficial effects of plasma-functionalization on the fibroblast attachment are pronounced enough to alter the fibroblast response in the long term.

Taken together, the results of our cell culture experiments revealed a transient impact of plasma-treated abutment surfaces on GF morphogenesis, and a higher sensitivity of AO to surface topography versus plasma-induced surface alterations. However, the effects of plasma-functionalization on the GF response as well as the effects of surface topography on the AO response were both restricted to cell morphogenesis. Neither the metabolic activity nor cell proliferation of GF and AO showed a significant dependency on the functionalization status or surface topography. As mentioned at the beginning, a recent own study and previous work from other groups demonstrated a positive correlation between cell shape/morphology and proliferation of human cells^43,60–63^. In detail, an increase in cell size led to an increase in cell proliferation in the aforementioned investigations. The lack in correlation between cell shape and proliferation in the present work might be explained by the finding that cell size and proliferation are not correlated linearly. Rather, changes in cell proliferation occur if a critical cell size is exceeded^43,60,61,63^. In this context, our previous results suggest that a critical AO size of about 3000–4000 µm^2^ is required to trigger osteoblast proliferation on implant surfaces^43^. The cell area detected in the present study for AO is however twice lower than aforementioned putative cell-innate proliferation stimulus. Based on these findings, it seems plausible to assume that the implant surface-dependent differences in AO morphology in the present work were not strong enough to trigger a change in proliferation behaviour. Even though such a correlation between cell shape/morphology and proliferation has not yet been reported for GF, the same mechanism could be here responsible for the week and transient effect of plasma-treated surfaces on GF proliferation. In order to substantiate these findings further studies in this context should consider primary human cells from a variety of donors.

## Conclusions

The current study demonstrates that oxygen plasma increases surface wettability of titanium- and zirconia-based implant biomaterials depending on the surface topography and initial wettability prior to functionalization. The plasma-induced wetting effect was thereby stronger on the rough implant surfaces when compared to the smooth counterparts. Hence, oxygen plasma-functionalization of implant surfaces represents a potential chairside modification method to increase the wettability of hydrophobic bone implants with optimized microroughness. Thinking one step ahead, the prospective consideration of plasma-responsive implant surface features in biomaterial design may enable tailoring implant surface characteristics towards tissue-optimized biomaterials for improved integration. This idea is supported by the results of our biological evaluation with primary human osteoblasts and gingival fibroblasts that revealed oxygen-plasma as cytocompatible functionalization method for titanium- and zirconia-based surfaces. In this context, we further demonstrated that on smooth surfaces GF were sensitive to the plasma-induced implant surface changes, albeit the cell response was only transient and restricted to the initial morphogenesis. The latter does, however, not preclude the possibility to develop implant surfaces that may control target cell behaviour via cell morphology upon plasma-functionalization.

## Materials and methods

### Implant materials and surface treatment

Biomaterial discs (20 mm in diameter, 1.5 mm in thickness) for cell culture experiments were made of alumina-toughened zirconia (ATZ; ATZ BIO-HIP®, Metoxit, Thayngen, Switzerland), yttria-stabilized tetragonal zirconia polycrystals (Y-TZP; TZP-A BIO-HIP®, Metoxit) and titanium (Ti; grade 4 titanium, Nobel Biocare, Kloten, Switzerland). These biomaterials represent three available materials that are successfully applied in oral implantology at present. Out of the multitude of optimized oral implant surfaces for enhanced osseointegration, we used the following clinically well-documented surface modifications for AO culture: (i) the microporous ZircaPore® surface layer on ATZ and (ii) on Y-TZP, and (iii) the TiUnite® surface created by electrochemical anodization of machined titanium discs resulting in a porous topography of the respective implants. Surfaces intended for GF culture were not further processed after the machining process to preserve the machined surface topography—as it is generally the case for surfaces of the transgingival implant part—and included the aforementioned material groups (i) ATZ, (ii) Y-TZP and (iii) Ti. All discs were sterilized by low-temperature hydrogen peroxide gas plasma sterilization (STERRAD 100/100S, Advanced Sterilization Products (A.S.P), Johnson & Johnson Medical, Irvine, USA) and stored under sterile conditions until usage. For plasma-functionalization the discs were exposed for 10 min to oxygen plasma in a Zepto Plasmacleaner (Diener Electronic, Ebhausen, Germany). For this, the sample chamber was evacuated and subsequently backfilled with oxygen gas (system pressure: 1 mbar, gas purity > 99.5%). The plasma-functionalized discs were used for surface characterization or cell culture experiments, and are hereinafter referred to as ATZ_AO_p/ ATZ_GF_p, Y-TZP_AO_p/ Y-TZP_GF_p and Ti_AO_p/ Ti_GF_p. Sterilized discs without further plasma-functionalization served as untreated control groups and are indicated as ATZ_AO/ ATZ_GF, Y-TZP_AO/ Y-TZP_GF and Ti_AO/ Ti_GF.

### Surface characterization

Surface topography of the implant materials was examined using a JSM-IT100 scanning electron microscope (SEM) (Jeol, Freising, Germany) with the backscattered electron imaging mode and an accelerated voltage of 15.00 kV after sputter coating with gold–palladium for 60 s at 60 mA (SCD050; Balzers, Liechtenstein).

The topography was characterized quantitatively in three dimensions by light interferometry (IFM) (n = 4 per group) as described earlier^42,43^. The measuring area of the interferometer (MicroXam 100 h; Phaseshift, Tucson, USA) was set to 260 × 200 μm. Before parameter calculation, a digital (Gaussian) filter of 50 × 50 μm was applied to remove errors of form and waviness. All surface parameters were calculated according to ISO 25178. To characterize the surface amplitude, the parameters S_a_, S_q_, S_z_ and S_sk_ were calculated (for parameter description see^64^). The S_a_-value describes the arithmetical mean height in μm and S_q_ represents the root-mean-square roughness—or in other words—the standard deviation of S_a_. S_z_ is the ten-point height of the surface topography and measures the average value of absolute surface disparities, whereas S_sk_ describes the skewness of the surface height distributions above/below a mean plane. S_sk_ adopts negative values if the surface exhibits more valleys than peaks while positive values indicate the opposite. Investigated hybrid parameters were S_dr_, which describes the surface enlargement compared to a totally flat reference area in % and S_dq_ which measures if the surface has any slopes. S_dq_ increases with increasing number of slopes and is reduced for smooth surfaces. The texture aspect ratio (S_tr_) is a spatial parameter that calculates the topographic texture pattern and adopts values between 0 and 1, whereas small values indicate strong anisotropy and large values indicate uniform texture aspect in all directions. S_pd_ and S_pc_ as feature parameters represent the number of peaks per area and the peak curvature, respectively. High values for S_pc_ indicate a pointed shape of the peaks whereas low values reflect rounded peaks.

Surface wettability of plasma-functionalized and control surfaces was investigated by measurement of the static contact angles (n = 8 per group) using a Dataphysics OCA 10 optical contact angle measuring system (Dataphysics GmbH, Filderstadt, Germany) in the sessile drop mode. For the measurement, distilled water was dispensed at a rate of 0.5 µl/s on the biomaterial surfaces. The static contact angles of 2 µl water droplets were then analysed 10 s after dispensing the droplets onto the surfaces with the DataPhysics SCA20 software (version 4.1.12, Dataphysics GmbH).

### Cell culture

Alveolar bone osteoblasts (AO) and gingival fibroblasts (GF) were prepared from bone and soft tissue explants obtained during dento-alveolar surgery. The collection and usage of the primary human osteoblasts and fibroblasts for scientific purposes was approved by the Ethics Committee of the Albert-Ludwigs-University, Freiburg, Germany (vote Nr. 411/08_121010). Informed consent was given by the patients. Research was performed in accordance with relevant guidelines and regulations. Both cell types were cultured in Dulbecco's Modified Eagle's Medium (Life Technologies, Darmstadt, Germany) supplemented with 1% (w/v) glutamine (Life Technologies), 10% (w/v) foetal calf serum (Biochrom AG, Berlin, Germany) and 0.2% (w/v) Kanamycin (Sigma-Aldrich, Taufkirchen, Germany). The cells were maintained in a humidified 37 °C incubator with 5% CO_2_. The osteoblastic phenotype of the isolated cells was verified by the extracellular matrix (ECM) mineralization potential of confluent cell cultures at day 28 as described by Perpétuo et al.^65^. Immunofluorescent staining of vimentin, a fibroblast specific marker^66,67^, was used to verify the fibroblastic phenotype. Osteoblasts and fibroblasts were passaged after reaching a confluence of approximately 80%. All experiments in this investigation were carried out with osteoblasts of passages 5 and 6 and fibroblasts of passages 9 and 10. For the cell culture on the biomaterial disks 1 × 10^4^ cells/ml were seeded per disk in a 12-well plate (cell density of 0.26 × 10^4^/cm^2^).

#### Cell adhesion and morphogenesis

Cell adhesion and morphogenesis on functionalized and non-functionalized biomaterial surfaces were examined by indirect immunofluorescence of vinculin and phalloidin-labeling of actin at days 1, 3 and 7 of cell culture. For fluorescence microscopy, cells were fixed with 4% formaldehyde in phosphate-buffered saline (PBS; Invitrogen, Karlsruhe, Germany) for 20 min at room temperature. Samples for immunocytochemistry were treated with 2% (w/v) bovine serum albumin (Sigma-Aldrich) in PBS and 0.2% TritonX-100 (Sigma-Aldrich) in PBS for 15 min and 2% (w/v) BSA in PBS for further 15 min. Thereafter, indirect immunofluorescence was performed by incubating the cells for one hour with mouse anti-vinculin (1:100; Abcam, Cambridge, UK) followed by incubation with AlexaFluor 488-conjugated goat anti-mouse IgG (1:200; Invitrogen) for one hour. Actin labelling was performed by incubating the samples for 30 min with phalloidin conjugate (TexasRed™-X Phalloidin, ThermoFisher, Waltham, USA) diluted 1:40 in PBS containing 0.5% BSA (w/v). Nuclei were stained with 0.001% Hoechst 33342 (Invitrogen) in 0.5% BSA in PBS for 15 min.

Optical evaluation was performed with the fluorescence microscope Biozero BZ-8000 (KEYENCE, Neu-Isenburg, Germany). Quantitative morphometric analysis of fluorescence micrographs was carried out with the microscope Software BZ-II Analyzer (version 2.2, KEYENCE) after 1 and 3 days of cell culture on the biomaterials. Morphology of as many cells as possible (145 ≤ n ≤ 373, 2 independent experiments each with 3 biomaterial discs per subgroup) was analysed as described earlier^68^ by measuring cell area, cell perimeter, the major axis representing the long axis of the smallest rectangle drawn around the cell body and the minor axis, presenting the rectangle width. To gain information about the cell shape on the different biomaterial surfaces, we further calculated a panel of shape descriptors derived from the aforementioned morphometric data according to Uynuk-Ool and co-workers^68^. These hybrid morphometric parameters included the cell´s aspect ratio (major axis/minor axis), cell roundness (4 × cell area/(π × major axis)^2^) and circularity (4 π × cell area/perimeter^2^). High values for the aspect ratio point to an elongated cell shape, whereas a high roundness parameter indicates a high degree of cell roundness. The parameter circularity describes the change from a circle with a large number of protrusions into a circle without protrusions, i.e., specifies whether cellular protrusions are formed or not. A high value indicates the absence of cellular protrusions.

#### Cell metabolism and viability

The cell metabolism of AO and GF on the plasma-functionalized and untreated test surfaces was analysed by using the alamarBlue® (AB) Cell Viability Assay (MorphoSys AbD, Düsseldorf, Germany). The AB reagent is a resazurin-based solution that is reduced in the mitochondrial respiratory chain to fluorescent resorufin which is released from the cell into the culture medium and can be quantified by fluorometry. The amount of resorufin in the culture medium gives information on the metabolic activity and viability of the cells. For this, the cell culture medium was removed after 1, 3 and 7 days of culture on the test surfaces and replaced with medium containing 10% (w/v) AB reagent. AO and GF were then incubated with the AB containing culture medium for 2 h at 37 °C (n = 6 per group, 2 independent experiments each with 3 biomaterial discs per subgroup). Afterwards the culture medium was analysed by measuring the fluorescence according to the manufacturer’s instructions. The percentage of AB reduction in the samples was calculated by application of the formula $percentage\;of\;alamarBlue\;reduction = \frac{FI 590\;of\;sample - FI 590\;of\;blank\;control}{{FI 590\;of\;100\% \;reduced\;alamarBlue - FI 590\;of\;blank\;control}}*100$ with FI 590 describing the fluorescent intensity at 590 nm emission. The blank control consisted of culture medium containing 10% (w/v) AB reagent. The 100% reduced AB reference (high control) was produced according to the manufacturer’s protocol by autoclaving culture medium with 10% (w/v) AB reagent for 15 min.

### Cell proliferation

To examine the cell proliferation of AO and GF at days 1, 3 and 7 of cell culture, nuclei of the cells were visualized with Hoechst 33342 as described above. The total cell number was determined by counting cell nuclei with the Software BZ Analyzer II at predefined areas on 6 biomaterial discs per subgroup (2 independent experiments).

### Statistical analysis

Differences between the test groups were examined for statistical significance using a one-way ANOVA followed by a Tukey´s HSD post hoc test for normally distributed data (according to the Shapiro–Wilk-test for normality) and a Kruskal–Wallis ANOVA followed by a Dunn´s post hoc test for non-normally distributed data. Data was investigated for possible correlations between different parameters by application of the Pearson´s test for correlation combined with a two-tailed t-test. The Pearson´s test for correlation provides values ranging from − 1 to 1, whereby values close to − 1 suggest an inverse correlation, values around 0 reject any correlation and values close to 1 point at a strong correlation between two parameters. All analyses were conducted with the software GraphPad Prism 9 (version 9.0.2, GraphPad Software, San Diego, U.S.A.).

## Supplementary Information

Supplementary Tables.

## Data Availability

The datasets generated during and/or analysed during the current study are available from the corresponding author on reasonable request.

## References

[1] Adell R, Lekholm U, Rockler B, Brånemark P-I (1981). A 15-year study of osseointegrated implants in the treatment of the edentulous jaw. Int. J. Oral Surg..

[2] Rompen E, Domken O, Degidi M, Pontes AEF, Piattelli A (2006). The effect of material characteristics, of surface topography and of implant components and connections on soft tissue integration: A literature review. Clin. Oral Implants Res..

[3] Rupp F, Liang L, Geis-Gerstorfer J, Scheideler L, Hüttig F (2018). Surface characteristics of dental implants: A review. Dent. Mater..

[4] Wennerberg A, Albrektsson T (2009). Effects of titanium surface topography on bone integration: A systematic review. Clin. Oral Implants Res..

[5] Teughels W, van Assche N, Sliepen I, Quirynen M (2006). Effect of material characteristics and/or surface topography on biofilm development. Clin. Oral Implants Res..

[6] Köunönen M, Hormia M, Kivilahti J, Hautaniemi J, Thesleff I (1992). Effect of surface processing on the attachment, orientation, and proliferation of human gingival fibroblasts on titanium. J. Biomed. Mater. Res..

[7] Kunzler TP, Drobek T, Schuler M, Spencer ND (2007). Systematic study of osteoblast and fibroblast response to roughness by means of surface-morphology gradients. Biomaterials.

[8] Sculean A, Gruber R, Bosshardt DD (2014). Soft tissue wound healing around teeth and dental implants. J. Clin. Periodontol..

[9] Lee J-H (2017). Non-thermal atmospheric pressure plasma functionalized dental implant for enhancement of bacterial resistance and osseointegration. Dent. Mater..

[10] Mandracci P, Mussano F, Rivolo P, Carossa S (2016). Surface treatments and functional coatings for biocompatibility improvement and bacterial adhesion reduction in dental implantology. Coatings.

[11] Henningsen A (2018). Photofunctionalization and non-thermal plasma activation of titanium surfaces. Clin. Oral Invest..

[12] Hoffmann C, Berganza C, Zhang J (2013). Cold atmospheric plasma: Methods of production and application in dentistry and oncology. Med. Gas Res..

[13] Cha S, Park Y-S (2014). Plasma in dentistry. Clin. Plasma Med..

[14] Giro G (2013). Osseointegration assessment of chairside argon-based nonthermal plasma-treated Ca-P coated dental implants. J. Biomed. Mater. Res. A.

[15] Monetta T, Bellucci F (2014). Strong and durable antibacterial effect of titanium treated in Rf oxygen plasma: Preliminary results. Plasma Chem. Plasma Process..

[16] Ibis F, Oflaz H, Ercan UK (2016). Biofilm inactivation and prevention on common implant material surfaces by nonthermal DBD plasma treatment. Plasma Med..

[17] Yoo E-M (2015). The study on inhibition of planktonic bacterial growth by non-thermal atmospheric pressure plasma jet treated surfaces for dental application. J. Biomed. Nanotechnol..

[18] Lee M-J (2019). The antibacterial effect of non-thermal atmospheric pressure plasma treatment of titanium surfaces according to the bacterial wall structure. Sci. Rep..

[19] Koban I (2011). Antimicrobial efficacy of non-thermal plasma in comparison to chlorhexidine against dental biofilms on titanium discs in vitro–proof of principle experiment. J. Clin. Periodontol..

[20] Wang L (2020). Bioactive effects of low-temperature argon–oxygen plasma on a titanium implant surface. ACS Omega.

[21] Duske K (2012). Atmospheric plasma enhances wettability and cell spreading on dental implant metals. J. Clin. Periodontol..

[22] González-Blanco C (2019). Human osteoblast cell behaviour on titanium discs treated with argon plasma. Materials.

[23] Wu C-C, Wei C-K, Ho C-C, Ding S-J (2015). Enhanced hydrophilicity and biocompatibility of dental zirconia ceramics by oxygen plasma treatment. Materials.

[24] Seon GM (2015). Titanium surface modification by using microwave-induced argon plasma in various conditions to enhance osteoblast biocompatibility. Biomater. Res..

[25] Shibata Y, Hosaka M, Kawai H, Miyazaki T (2002). Glow discharge plasma treatment of titanium plates enhances adhesion of osteoblast-like cells to the plates through the integrin-mediated mechanism. Int. J. Oral Maxillofac. Surg..

[26] Zheng Z (2020). Effects of novel non-thermal atmospheric plasma treatment of titanium on physical and biological improvements and in vivo osseointegration in rats. Sci. Rep..

[27] Smeets R (2019). Influence of ultraviolet irradiation and cold atmospheric pressure plasma on zirconia surfaces: An in vitro study. Int. J. Oral Maxillofac. Surg..

[28] Swart KM (1992). Short-term plasma-cleaning treatments enhance in vitro osteoblast attachment to titanium. J. Oral Implantol..

[29] Guo L (2019). Cytocompatibility of titanium, zirconia and modified PEEK after surface treatment using UV light or non-thermal plasma. Int. J. Mol. Sci..

[30] Lee J-H, Kim Y-H, Choi E-H, Kim K-M, Kim K-N (2015). Air atmospheric-pressure plasma-jet treatment enhances the attachment of human gingival fibroblasts for early peri-implant soft tissue seals on titanium dental implant abutments. Acta Odontol. Scand..

[31] Jeong W-S, Kwon J-S, Choi E-H, Kim K-M (2018). The effects of non-thermal atmospheric pressure plasma treated titanium surface on behaviors of oral soft tissue cells. Sci. Rep..

[32] Canullo L, Cassinelli C, Götz W, Tarnow D (2013). Plasma of argon accelerates murine fibroblast adhesion in early stages of titanium disk colonization. Int. J. Oral Maxillofac. Implants.

[33] Czekanska EM, Stoddart MJ, Richards RG, Hayes JS (2012). In search of an osteoblast cell model for in vitro research. Eur. Cell Mater..

[34] Lohmann CH (2000). Maturation state determines the response of osteogenic cells to surface roughness and 1,25-dihydroxyvitamin D3. J. Bone Miner. Res..

[35] Saldaña L, Bensiamar F, Boré A, Vilaboa N (2011). In search of representative models of human bone-forming cells for cytocompatibility studies. Acta Biomater..

[36] Czekanska EM, Stoddart MJ, Ralphs JR, Richards RG, Hayes JS (2014). A phenotypic comparison of osteoblast cell lines versus human primary osteoblasts for biomaterials testing. J. Biomed. Mater. Res. A.

[37] Altmann B (2013). Distinct cell functions of osteoblasts on UV-functionalized titanium- and zirconia-based implant materials are modulated by surface topography. Tissue Eng. Part C Methods.

[38] Li S (2012). Surface characteristics and biocompatibility of sandblasted and acid-etched titanium surface modified by ultraviolet irradiation: An in vitro study. J. Biomed. Mater. Res. Part B Appl. Biomater..

[39] Li B (2014). Improvement of biological properties of titanium by anodic oxidation and ultraviolet irradiation. Appl. Surf. Sci..

[40] Anselme K (2000). Osteoblast adhesion on biomaterials. Biomaterials.

[41] Anselme K, Ponche A, Bigerelle M (2010). Relative influence of surface topography and surface chemistry on cell response to bone implant materials. Part 2: biological aspects. Proc. Inst. Mech. Eng. H..

[42] Altmann B (2017). Assessment of novel long-lasting ceria-stabilized zirconia-based ceramics with different surface topographies as implant materials. Adv. Funct. Mater..

[43] Rabel K (2020). Controlling osteoblast morphology and proliferation via surface micro-topographies of implant biomaterials. Sci. Rep..

[44] Law K-Y (2014). Definitions for hydrophilicity, hydrophobicity, and superhydrophobicity: Getting the basics right. J. Phys. Chem. Lett..

[45] Grischke J, Eberhard J, Stiesch M (2016). Antimicrobial dental implant functionalization strategies—A systematic review. Dent. Mater. J..

[46] Canullo L, Genova T, Wang H-L, Carossa S, Mussano F (2017). Plasma of argon increases cell attachment and bacterial decontamination on different implant surfaces. Int. J. Oral Maxillofac. Surg..

[47] Rupp F, Scheideler L, Rehbein D, Axmann D, Geis-Gerstorfer J (2004). Roughness induced dynamic changes of wettability of acid etched titanium implant modifications. Biomaterials.

[48] Rupp F (2006). Enhancing surface free energy and hydrophilicity through chemical modification of microstructured titanium implant surfaces. J. Biomed. Mater. Res. A.

[49] Murakami D, Jinnai H, Takahara A (2014). Wetting transition from the Cassie–Baxter state to the Wenzel state on textured polymer surfaces. Langmuir.

[50] Cassie AB, Baxter S (1944). Wettability of porous surfaces. Trans. Faraday Soc..

[51] Henningsen A (2018). Changes in surface characteristics of titanium and zirconia after surface treatment with ultraviolet light or non-thermal plasma. Eur. J. Oral Sci..

[52] Shon W-J (2014). Peri-implant bone formation of non-thermal atmospheric pressure plasma-treated zirconia implants with different surface roughness in rabbit tibiae. Clin. Oral Implants Res..

[53] Coelho PG (2012). Argon-based atmospheric pressure plasma enhances early bone response to rough titanium surfaces. J. Biomed. Mater. Res. A.

[54] Noro A, Kaneko M, Murata I, Yoshinari M (2013). Influence of surface topography and surface physicochemistry on wettability of zirconia (tetragonal zirconia polycrystal). J. Biomed. Mater. Res. Part B Appl. Biomater..

[55] Ventre M, Natale CF, Rianna C, Netti PA (2014). Topographic cell instructive patterns to control cell adhesion, polarization and migration. J. R. Soc. Interface.

[56] Koban I (2011). Atmospheric Plasma Enhances Wettability and Osteoblast Spreading on Dentin In Vitro: Proof-of-Principle. Plasma Process. Polym..

[57] Humphries JD (2007). Vinculin controls focal adhesion formation by direct interactions with talin and actin. J. Cell Biol..

[58] Woodruff MA, Jones P, Farrar D, Grant DM, Scotchford CA (2007). Human osteoblast cell spreading and vinculin expression upon biomaterial surfaces. J. Mol. Histol..

[59] Yamano S (2011). The influence of different implant materials on human gingival fibroblast morphology, proliferation, and gene expression. Int. J. Oral Maxillofac. Surg..

[60] Dike LE (1999). Geometric control of switching between growth, apoptosis, and differentiation during angiogenesis using micropatterned substrates. Vitro Cell Dev. Biol. Anim..

[61] Chen CS, Mrksich M, Huang S, Whitesides GM, Ingber DE (1997). Geometric control of cell life and death. Science.

[62] Singhvi R (1994). Engineering cell shape and function. Science.

[63] Thakar RG (2009). Cell-shape regulation of smooth muscle cell proliferation. Biophys. J..

[64] Waikar RA, Guo YB (2008). A comprehensive characterization of 3D surface topography induced by hard turning versus grinding. J. Mater. Process. Technol..

[65] Perpétuo IP, Bourne LE, Orriss IR, Idris AI (2019). Bone Research Protocols.

[66] Przekora A, Zarnowski T, Ginalska G (2017). A simple and effective protocol for fast isolation of human Tenon’s fibroblasts from a single trabeculectomy biopsy–a comparison of cell behaviour in different culture media. Cell Mol. Biol. Lett..

[67] Nejaddehbashi F (2019). Isolating human dermal fibroblasts using serial explant culture. Stem Cell Investig..

[68] Uynuk-Ool T (2017). The geometrical shape of mesenchymal stromal cells measured by quantitative shape descriptors is determined by the stiffness of the biomaterial and by cyclic tensile forces. J. Tissue Eng. Regen. Med..

