# Supplementary material for: Human osteoblast and fibroblast response to oral implant biomaterials functionalized with non-thermal oxygen plasma
Source: Sci Rep. 2021 Aug 27;11:17302. doi: 10.1038/s41598-021-96526-x (PMC8397744; doi:10.1038/s41598-021-96526-x)
Supplement: Supplementary file 1 — Supplementary Tables. [file 41598_2021_96526_MOESM1_ESM.pdf]

Human osteoblast and fibroblast response to oral implant biomaterials functionalized with non-thermal oxygen plasma

Kerstin Rabel, DMD[1*], Ralf-Joachim Kohal, PhD, DMD[1], Thorsten Steinberg, PhD[2], Bernd Rolauffs[3], Erik Adolfsson, PhD[4], Brigitte Altmann, PhD[1, 5]

1 Department of Prosthetic Dentistry, Center for Dental Medicine, Medical Center - University of Freiburg, Faculty of Medicine, University of Freiburg, Hugstetterstr. 55, 79106 Freiburg, Germany

2 Department of Oral Biotechnology, Center for Dental Medicine, Medical Center - University of Freiburg, Faculty of Medicine, University of Freiburg, Hugstetterstr. 55, 79106 Freiburg, Germany

3 G.E.R.N. Center for Tissue Replacement, Regeneration & Neogenesis, Department of Orthopedics and Trauma Surgery, Medical Center - University of Freiburg, Faculty of Medicine, University of Freiburg, Engesserstr. 4, 79108 Freiburg, Germany.

4 RISE IVF AB, RISE Research Institutes of Sweden, Argongatan 30, 43153 Mölndal, Sweden

5 G.E.R.N. Center for Tissue Replacement, Regeneration & Neogenesis, Department of Prosthetic Dentistry, Medical Center - University of Freiburg, Faculty of Medicine, University of Freiburg, Engesserstr. 4, 79108 Freiburg, Germany

*Correspondence to: Kerstin Rabel,

kerstin.rabel@uniklinik-freiburg.de

Biomaterial	S_a [µm]	S_q [µm]	S_z [µm]	S_{sk} [µm]	S_{tr}	S_{dr} [%]	Sdq	Spd	Spc
**ATZ_AO**	0.68 ± 0.09	1.02 ± 0.19	14.05 ± 1.25	-0.54 ± 0.23	0.79 ± 0.06	26.17 ± 2.37	1.35 ± 0.16	0.002 ± 0.00	11.66 ± 5.05
**TZP_AO**	0.64 ± 0.02	1.06 ± 0.04	13.51 ± 0.12	-2.6 ± 0.04	0.77 ± 0.06	33.44 ± 0.66	1.72 ± 0.03	0.003 ± 0.00	12.29 ± 0.45
**Ti_AO**	1.3 ± 0.06	1.55 ± 0.06	10.17 ± 0.47	0.57 ± 0.06	0.91 ± 0.00	57.14 ± 2.83	2.13 ± 0.08	0.01 ± 0.00	8.60 ± 0.78

Supplementary Table S1: Surface parameters of the biomaterial samples for AO. Data show mean values (n=4) ± SEM.

Biomaterial	S_a [µm]	S_q [µm]	S_z [µm]	S_{sk} [µm]	S_{tr}	S_{dr} [%]	Sdq	Spd	Spc
**ATZ_GF**	0.13 ± 0.00	0.18 ± 0.00	2.84 ± 0.47	-2.08 ± 0.14	0.73 ± 0.04	1.36 ± 0.05	0.18 ± 0.01	0.003 ± 0.00	0.26 ± 0.05
**TZP_GF**	0.17 ± 0.02	0.24 ± 0.03	3.19 ± 0.72	-1.50 ± 0.22	0.84 ± 0.02	2.70 ± 0.40	0.27 ± 0.04	0.004 ± 0.00	0.66 ± 0.29
**Ti_GF**	0.14 ± 0.01	0.19 ± 0.03	3.02 ± 1.06	0.82 ± 0.88	0.12 ± 0.05	1.23 ± 0.19	0.18 ± 0.03	0.006 ± 0.00	0.66 ± 0.12

Supplementary Table S2: Surface parameters of the biomaterial samples for GF. Data show mean values (n=4) ± SEM.

	Not functionalized ± SEM	Functionalized ± SEM
**ATZ_AO**	75.65 ± 6,80	12.90 ± 0.59
**TZP_AO**	96.04 ± 3.29	0.78 ± 0.51
**Ti_AO**	97.43 ± 1.60	0.00 ± 0.00
**ATZ_GF**	72.51 ± 5.81	15.71 ± 0.96
**TZP_GF**	63.86 ± 4.41	12.34 ± 0.40
**Ti_GF**	65.43 ± 3.94	7.94 ± 0.84

Supplementary Table S3: Contact angles of plasma-functionalized and control surfaces for AO and GF. Data show mean values (n=8) ± SEM.

Biomaterial	cell area [µm²]		perimeter [µm]		aspect ratio		roundness		circularity	
	day 1	day 3	day 1	day 3	day 1	day 3	day 1	day 3	day 1	day 3
**ATZ_AO**	1813±72	1755±96	204±6	201±7	2.06±0.07	2.23±0.09	0.16±0.00	0.15±0.00	0.58±0.01	0.58±0.01
**ATZ_AO_p**	1724±78	1564±83	204±6	180±6	2.34±0.09	2.01±0.07	0.14±0.00	0.16±0.00	0.55±0.01	0.62±0.01
**Y-TZP_AO**	1726±59	1606±67	206±5	222±8	2.39±0.08	2.53±0.09	0.14±0.00	0.13±0.00	0.55±0.01	0.48±0.01
**Y-TZP_AO_p**	1775±64	1810±84	207±5	213±7	2.41±0.08	2.55±0.10	0.14±0.00	0.13±0.00	0.55±0.01	0.54±0.01
**Ti_AO**	1701±44	1581±72	241±6	224±9	2.59±0.08	2.62±0.09	0.11±0.00	0.11±0.00	0.43±0.01	0.46±0.01
**Ti_AO_p**	1826±47	1440±63	236±5	216±8	2.53±0.07	2.7±0.10	0.12±0.00	0.12±0.00	0.47±0.01	0.49±0.02

Supplementary Table S4: Morphometric data of AO morphology after 1 and 3 days. Data are listed as mean values ± SEM (194<n<373).

Biomaterial	cell area [µm²]		perimeter [µm]		aspect ratio		roundness		circularity	
	day 1	day 3	day 1	day 3	day 1	day 3	day 1	day 3	day 1	day 3
**ATZ_GF**	2435±130	2526±195	309±13	283±15	3.82±0.20	3.35±0.18	0.09±0.01	0.10±0.01	0.41±0.02	0.44±0.02
**ATZ_GF_p**	2126±111	2697±226	253±9	286±14	3.41±0.18	3.22±0.15	0.11±0.01	0.10±0.00	0.47±0.02	0.43±0.02
**Y-TZP_GF**	2597±156	1692±109	320±12	213±9	3.67±0.18	2.60±0.10	0.09±0.00	0.13±0.00	0.36±0.01	0.54±0.01
**Y-TZP_GF_p**	1881±73	2013±129	253±8	239±10	3.23±0.11	2.68±0.10	0.10±0.00	0.12±0.00	0.44±0.01	0.51±0.01
**Ti_GF**	2808±114	2098±109	348±10	271±10	4.13±0.16	3.65±0.14	0.07±0.00	0.11±0.01	0.33±0.01	0.44±0.01
**Ti_GF_p**	2372±119	1933±104	304±13	259±10	3.83±0.19	3.50±0.12	0.09±0.01	0.10±0.00	0.41±0.02	0.45±0.02

Supplementary Table S5: Morphometric data of GF morphology after 1 and 3 days. Data are listed as mean values ± SEM (145<n<300).

	AB reduction [%]			Cell number		
	day 1	day 3	day 7	day 1	day 3	day 7
**ATZ AO**	2.35±0.5	3.90±0.8	13.15±3.5	24±6	46±8	81±24
**ATZ AO_p**	2.04±0.3	3.42±0.5	11.42±2.0	16±2	65±10	105±34
**Y-TZP AO**	2.27±0.4	3.84±0.5	11.89±2.0	22±16	39±5	125±8
**Y-TZP AO_p**	2.00±0.3	3.23±0.4	10.91±1.6	26±3	51±8	107±19
**Ti AO**	2.37±0.5	3.83±0.7	10.01±2.0	33±3	51±6	101±14
**Ti AO_p**	1.93±0.3	3.31±0.3	9.51±1.5	29±3	45±6	147±16

Supplementary Table S6: Metabolic activity and number of attached AO. Data are listed as mean values ± SEM (n=6).

	AB reduction [%]			Cell number		
	day 1	day 3	day 7	day 1	day 3	day 7
**ATZ GF**	1.98±0.1	4.70±0.2	10.93±0.8	16±2	33±5	97±7
**ATZ GF_p**	1.6±0.1	4.22±0.2	9.53±0.4	26±4	33±6	67±8
**Y-TZP GF**	2.05±0.1	4.89±0.3	10.58±0.7	20±2	40±4	114±8
**Y-TZP GF_p**	1.63±0.1	4.21±0.1	9.36±0.2	20±2	37±5	90±13
**Ti GF**	1.88±0.1	4.42±0.3	9.80±0.7	23±2	42±5	131±12
**Ti GF_p**	1.61±0.1	4.30±0.3	9.69±0.3	20±3	51±9	70±8

Supplementary Table S7: Metabolic activity and number of attached GF. Data are listed as mean values ± SEM (n=6).
